# Exploring Autophagy Inducing Molecules: Targeting Diverse Pathways in Alzheimer's Disease Management

**DOI:** 10.1002/med.70013

**Published:** 2025-09-29

**Authors:** Baljinder Singh, Shubham Mahajan, Sadikalmahdi Abdella, Rehan Khan, Sanjay Garg

**Affiliations:** ^1^ Center for Pharmaceutical Innovation, Clinical and Health Sciences University of South Australia Adelaide Australia; ^2^ Chemical Biology Unit Institute of Nano Science and Technology Mohali India

**Keywords:** autophagy inducers, neurodegenerative disorders, phytoconstituents, repurposed molecules, senile dementia

## Abstract

Neurodegenerative disorders, including Alzheimer's disease (AD), impose a significant burden on society due to their progressive nature and the associated healthcare costs. Autophagy, a vital cellular degradation process, has emerged as a promising therapeutic target in these disorders. This review aims to provide a comprehensive overview of autophagy's role in neurodegenerative diseases, focusing on AD. The pathogenesis of AD involves the accumulation of misfolded proteins, such as beta‐amyloid (Aβ) and tau, leading to neuronal dysfunction and cognitive impairment. Autophagy can be crucial in clearing these protein aggregates and maintaining cellular homeostasis. Nevertheless, autophagic dysregulation and mitochondrial dysfunction contribute to further progression of neurodegeneration. Furthermore, recent studies have demonstrated the therapeutic potential of several plant‐based phytoconstituents and repurposed molecules that modulate autophagy. These compounds target both mTOR‐dependent and independent pathways, highlighting their potential to alleviate disease pathology. This review aims to pave the way for future research and development in this field.

## 1 Introduction

Neurodegenerative disorders are a significant burden on society, characterized by the progressive deterioration of neurons in the central nervous system (CNS). These modern‐day incongruities, including Parkinson's disease (PD), Alzheimer's disease (AD), migraine, meningitis, and schizophrenia, are on the rise due to various factors such as genetic abnormalities, congenital conditions, malnutrition, stressful lifestyles, and environmental issues [1]. Among these disorders, dementia, particularly dementia due to AD, imposes a substantial economic burden, which is escalating rapidly. The World Health Organization (WHO) reported that approximately 5.8 million people in America are affected by AD‐related dementia, costing around $290 billion. Alarmingly, these numbers are projected to reach 14 million individuals and $1.1 trillion by 2050 [2]. The mortality rate due to dementia has also seen a sharp increase, with a surge of almost 145%(Year)compared to the year 2000.

The pathogenesis of AD involves the continuous progression of neuronal deterioration causing cognitive impairment and memory loss [3]. Alois Alzheimer's pioneering work in dementia defined AD as “senile dementia”. However, it is now understood as age‐related neurodegeneration, characterized by the accumulation of misfolded beta‐amyloid proteins and neurofibrillary tangles within neurons [4].

Amyloid precursor protein (APP), present on the surface of the endoplasmic reticulum, undergoes proteolytic cleavage to produce Aβ proteins, which can accumulate both intracellularly and extracellularly [5]. Two pathways, namely the amyloidogenic and non‐amyloidogenic pathways, govern the cleavage of these transmembrane proteins [6]. While Aβ_40_ and Aβ_42_ are normal forms present in the brain, the AD brain exhibits an increased accumulation of insoluble Aβ_42_ [7]. Additionally, the hyperphosphorylation of tau protein leads to the formation of insoluble tangle filaments and the disintegration of microtubules, further contributing to neuronal dysfunction [8, 9].

The clearance of these unwanted and damaged protein aggregates, including misfolded Aβ and tau, is mediated by autophagy, a cellular degradative‐catabolic process crucial for maintaining cellular homeostasis [10]. Autophagy plays a prominent role in removing intracellular and extracellular protein aggregates and damaged organelles. Among its various modes, macroautophagy is the most common form, followed by chaperone‐mediated autophagy and microautophagy [11]. Notably, the dysregulation of autophagy and mitochondrial dysfunction contributes to the pathogenesis of age‐related neurodegenerative diseases like Alzheimer's disease [12].

The clinical evidence suggests that autophagy holds great promise in managing Alzheimer‐related dementia. However, a better understanding of complex mechanisms underlying autophagy dysregulation in AD diseases opens avenues for developing effective treatments. This review aims to provide a comprehensive overview of autophagy in neurodegenerative diseases including AD, emphasizing the potential of therapeutics targeting autophagy especially plant‐derived compounds that hold the promise for future research and development. Additionally, we have also included some repurposed molecules used as autophagy modulators that can be used in dementia related to Alzheimer's.

## 2 Molecular Mechanisms

The direct evidence linking autophagy to Alzheimer's disease was first presented by Nixon and group, where the pathological evaluation revealed the presence of autophagic vacuoles, multilamellar bodies, and dense residuals in affected neuronal cells [13]. Subsequently in another study, further demonstration of the relationship between impaired autophagic vesicle clearance and the accumulation of these vesicles in AD neurons was illustrated [14]. While the endoplasmic reticulum and mitochondria have important correlations with autophagy, the phagophore is considered the initiation site of this process [15].

Regulation of autophagy mainly occurs through two pathways: the mammalian target of rapamycin (mTOR)‐dependent and mTOR‐independent pathways. The mTOR‐dependent pathway involves kinases such as ULK1 and VPS34, which create a pathway for autophagy regulation [16]. Akt‐mediated phosphorylation of mTOR inhibits autophagy, while inactivation of these kinases promotes autophagy [17]. Alternatively, AMPK activation can induce autophagy by inhibiting mTOR1 and promoting phagosome formation. Therapeutic strategies targeting these pathways, particularly inhibiting mTOR complex 1, hold promise in modulating autophagy [18].

**Figure 1:**
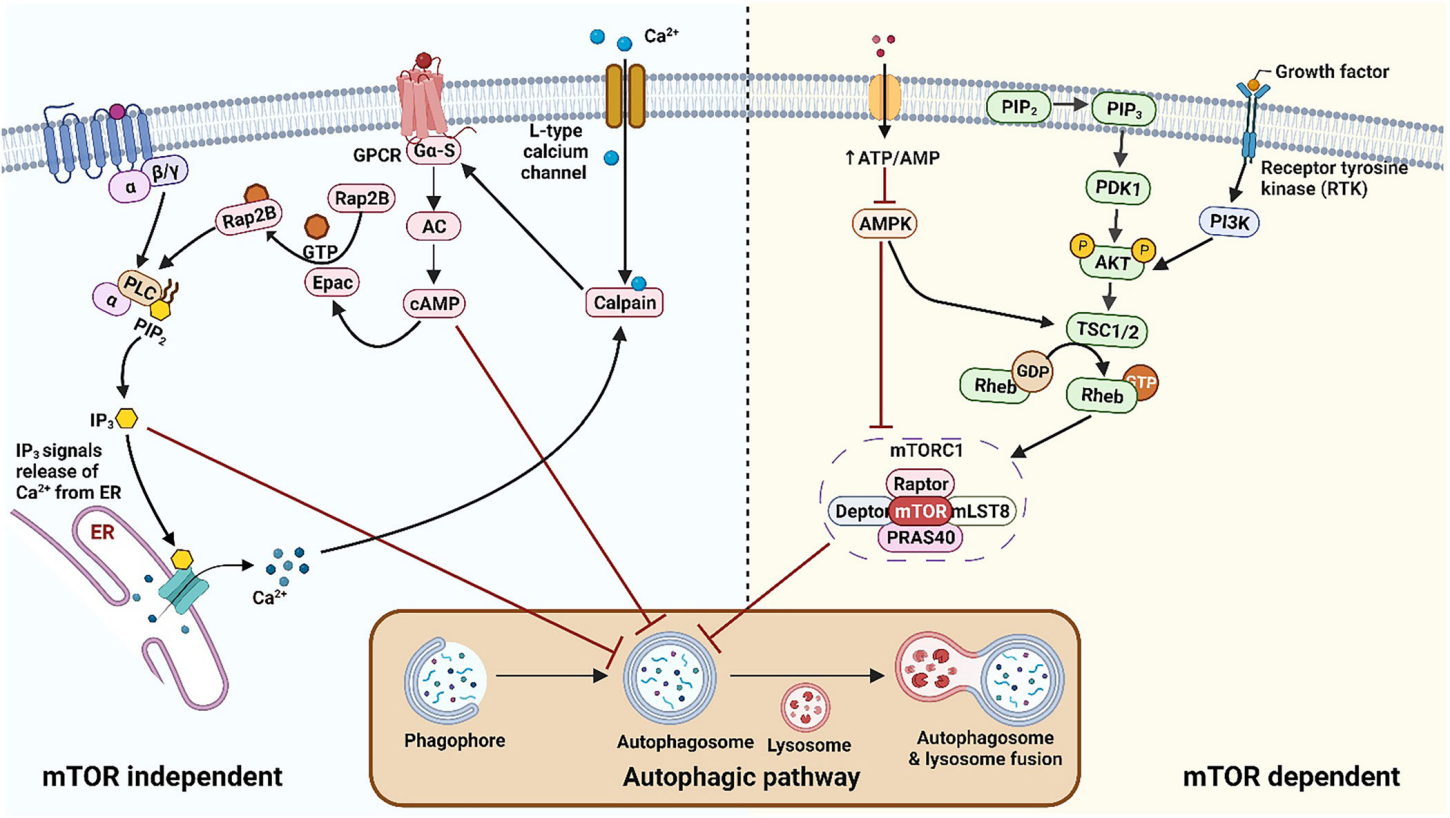
The depiction showing various m‐TOR dependent and m‐TOR independent autophagic pathways in Alzheimer's disease. “Created with Biorendor. com.” [Color figure can be viewed at wileyonlinelibrary.com]

In addition to the mTOR‐dependent pathway, independent pathways such as the inositol signaling pathway, calpain signaling pathway, and cAMP pathway have been explored as therapeutic targets for autophagy induction [17]. The inositol pathway, associated with glycolysis and mitochondrial functions, promotes cell growth, but decreased inositol levels can induce autophagy and facilitate the clearance of cellular debris [19]. Similarly, the Ca^2+^/calpain pathway, influenced by cytosolic calcium levels, affects cellular growth, and increased levels inhibit autophagy [20]. Finally, the cAMP pathway, governed by adenosine 3ʹ,5ʹ‐cyclic monophosphate, plays a crucial role in cell signal transduction and can induce autophagy through specific targets like protein kinase A and Epac [18]. Both these mTOR‐dependent and independent pathways are briefly presented in Figure 1 below.

Considering the potential of autophagy as a therapeutic target, extensive research has been focused on identifying compounds from medicinal plants with potent therapeutic activities and low toxicity. Polyphenols, alkaloids, terpenes, and terpenoids from various plant sources have shown promise in treating neurodegenerative diseases [21]. For instance, polyphenols like curcumin and resveratrol have demonstrated in vivo activity in inducing autophagy. Curcumin activates autophagy via the PI3K/Akt/mTOR pathway, while resveratrol induces autophagy through the AMPK/mTORC1 pathway [22, 23]. Additionally, compounds like quercetin, berberine, and Rg2 from ginseng, have also shown the potential to induce autophagy through specific pathways [24, 25, 26].

## 3 Role of Autophagy in Neuroglia

The neuroglia is a connective tissue present in the peripheral nervous system (PNS) and central nervous system (CNS) which is important for maintaining the homeostasis of the brain via different pathways. The glial cells of the CNS are classified into microglia, macroglia and myeloid lineage cells [27]. Autophagy is an important process in astrocytic cells to clear debris such as protein aggregates and other defective organelles. Astrocytes, meanwhile, have brain region‐specific functions such as calcium signaling at synapses, regulation of manganese concentrations in the brain, and regulation of the blood‐brain barrier, thus also referred to as housekeeping cells [28]. Although autophagy is a ubiquitous process and can be cell‐specific, the ability of astrocytes along with microglial cells to clear extracellular beta‐amyloid aggregates by an APOE‐dependent internalization process is crucial in AD [29]. A study by Simonovitch et al presented that the APOE4 knock‐in mice model had less autophagic clearance compared to the APOE3 model [30]. Similarly, astrocytes' insulin‐degrading enzyme (IDE) secretion is actively triggered via an autophagy‐dependent nonconventional pathway and influences extracellular Aβ degradation [31, 32]. Another pathway of neuroinflammation in AD can trigger the microglia and astrocytes to release anti‐ and proinflammatory cytokines leading to a decrease in mitochondrial integrity and an increase in reactive species, finally causing cell death [33, 34]. Furthermore, the microglial NLRP3‐inflammasome overexpression can cause excess production of inflammatory cytokines and neuronal death. A study by Cho et al confirmed this concept, showing that *ATG7* or *LC3* gene knockdown in mice model increased the NLRP3 inflammasome activity significantly [35]. In conclusion, data showed that microglial autophagy can promote cell survival by decreasing inflammasome activity, which has disease‐modifying potential.

Oligodendrocytes play an important role in the formation of myelin sheath, providing nutrition to the neurons, and signal transmission [36, 37]. NG2 cells, a subtype of oligodendrocytes show affinity towards Aβ42, which they degrade with the assistance of astrocytic projections. In a similar vein, Aber et al presented the potential role of the autophagic ability of oligodendrocytes using the *Atg5* or *Atg7* phenotype. The findings suggested that the abnormal thickening and accumulation of myelin sheath mimicked pathological conditions similar to AD [38]. The disruption in autophagic functioning may further lead to neurodegenerative disease progression [39].

Autophagic processes play a crucial role in clearing cellular debris, such as aggregated amyloid‐beta proteins and neurofibrillary tangles, from various neuroglial pathways. Disruption of autophagy can exacerbate Alzheimer's disease pathology. Our work has presented different phytoconstituents that can enhance disease outcomes by promoting autophagy.

## 4 Phytoconstituents in Autophagy

### 4.1 Polyphenols as Autophagy Inducer

Naturally occurring phytochemicals have been used in various conditions including in ayurveda and Chinese medicines. Their active constituents can part disease‐changing behavior through different mechanisms; however, clear pathways are yet to be discovered. These compounds derived from medicinal plants are summarized in Table 1 and show potential as autophagy modulators, offering a safer alternative for therapeutic intervention. Polyphenol compounds found abundantly in various plant sources, represent an innovative approach to targeting autophagy for AD. Emerging research suggests that polyphenols demonstrate the potential to modulate autophagy pathways. These natural compounds have shown in vivo activity, inducing autophagy through specific signaling pathways such as the PI3K/Akt/mTOR and AMPK/mTORC1 pathways. We have summarized a few examples from the literature working on similar lines.

**Table 1:** Plant‐based molecules belonging to different classes with the potential to modulate autophagy via different targeting pathways are summarized.

Molecule	Class	Targets/pathways investigated for autophagy	Activity
Curcumin	Polyphenol	PI3K/Akt/mTOR mediated [22], TFEB mediated, chaperone‐mediated [40]	↓Aβ load
Resveratrol	Polyphenol	↑LC3‐II, ↓p62 [41], Mitophagy (similar study) AMPK and mTOR mediated (similar study)	↓Aβ25–35‐induced toxicity ↓Aβ load
Hesperidin	Polyphenol	VDAC1 phosphorylation mediated [42] Aβ induced autophagy [43]	↓Aβ25–35‐induced apoptosis ↓ Aβ induced impairment in glucose metabolism
Hesperetin	Polyphenol	Aβ induced autophagy [43]	↓ Aβ induced impairment in glucose metabolism
Oxyresveratrol	Polyphenol	AMPK/ULK1/mTOR‐mediated [44]	↓Aβ load ↓γ‐secretase activity
Piceatannol	Polyphenol	TFEB/TFE3 pathway [45]	↓Aβ load ↑α‐secretase activity
Oleuropein aglycone	Polyphenol	AMPK/mTOR pathway [46]	↓Aβ load ↓ neuroinflammation
Hydroxytyrosol	Polyphenol	↓S6, p62; ↑Beclin‐1, LC3‐II/LC3‐I ratio [47]	↓Aβ toxicity
Isovitexin	Polyphenol	↑LC3II, Beclin‐1, Atg7 mediated [48]	↓Aβ load
Ferulic acid	Polyphenol	LGG‐1 mediated [49]	↓protein misfolding
Kaempferol	Polyphenol	↑Beclin‐1, LC3B‐II, AMBRA1; ↓p62 [50]	↓Aβ load
Rhapontigenin	Polyphenol	↑ULK1 phosphorylation at Ser555 [50]	↓Aβ load
Berberine	Alkaloid	↑LC3‐II, Beclin‐1, hVps34, Cathepsin‐D; ↓p62 levels [51] class III PI3K/beclin‐ 1 cascade [52]	↓Aβ load ↓Aβ25–35‐induced apoptosis ↓tau‐hyperphosphorylation
Galantamine	Alkaloid	α7nAChR and Akt inhibition mediated [53]	↓ Aβ1–42 neurotoxicity
DNLA (*Dendrobium nobile* Lindl alkaloid)	Alkaloid	↑ GFP‐LC3B [54]	↓Aβ25–35‐induced toxicity
Corynoxine	Alkaloid	LC3‐II [55]	↓Aβ load
Piperlongumine	Alkaloid	↑BCL2 phosphorylation at Ser70 [56]	↓Aβ toxicity ↑cognitive ability
Isorhynchophylline	Alkaloid	BECLIN‐1 mediated [57]	↓Aβ neurotoxicity ↓alpha‐synuclein
Neferine	Alkaloid	↓p62, LC3; ↑LC3‐II:LC3I [58]	↓Aβ load, HTT74, P301L tau, A53T α‐synuclein
Physostigmine	Alkaloid	↓p62/SQSTM1 [59]	↑cognitive ability
Gingko biloba extracts	extract	LC3‐II mediated [60]	↓Aβ load ↓Aβ_25‐35_‐induced toxicity
Ouabain	Glycoside	TFEB mediated [60]	↓tau‐hyperphosphorylation
cornel iridoid glycoside	Glycoside	GSK‐3β mediated [61]	↓Aβ_25‐35_‐induced toxicity ↓tau oligomer‐induced neurotoxicity
DMDD from *Averrhoa carambola*	Dione	Bax/Bcl‐2 mediated mitochondrial pathway [62]	↓Apoptosis related to Aβ_1‐42_
Icariin	Flavanol glycoside	BDNF/TrkB pathway activation [63]	↑cognitive ability via improved synaptic plasticity
GP‐17 (Gypenoside XVII)	Saponin	TFEB release from TFEB/14‐3‐3 complexes [64]	↓Aβ load via autophagy
Arctigenin	Lignan	PERK/eIF2α pathway inhibition and decreased BACE1 translationmTOR dependent autophagy [65]	↓Aβ production and increased clearance
Sulforaphane	Iso‐thiocyanate	ERK activation [66]	↑neuroprotective activity
Escin	Saponin	Autophagy activation via ATG7 mTOR and ERK signaling [67]	↓Aβ load via autophagy

In a study, Wang et al tested curcumin to halt the Alzheimer's neurodegeneration cascade by suppressing the PI3K/Akt/mTOR signaling pathway through autophagy augmentation. They performed an array of studies to investigate the autophagy‐inducing potential of curcumin using immunohistology and fluorescence analysis and western blot assays on LC3 cells in 2x transgenic AD mice. The results showed that curcumin efficiently improved cognitive impairments and attenuated Aβ generation in APP/PS1 2x transgenic AD mice. A significant decline in a cascade of P13K was observed along with depression in phosphorylated Akt and mTOR protein levels defining the anti‐Alzheimer activity. Hence, the current study establishes that curcumin efficiently blocked Aβ production parallel to augmented autophagy by downregulating PI3K/Akt/mTOR signaling pathways [22]. In a similar work, Ju‐Xian and team employed curcumin to trigger transcription factor EB (TFEB), which is involved in autophagy regulation and lysosomal biogenesis. They went on to synthesize and screen monocarbonyl analogs of curcumin as autophagy‐inducers in the neuroblastoma N2a cell mouse model. The observations depicted a dose‐dependent increase in the levels of LC3B‐II in the N2a cells vis‐a‐vis vehicle control, and C1 analogs increased the nuclear translocation of TFEB. Autolysosome formation, a key factor in autophagy was confirmed through in vitro, and in vivo studies. In conclusion, curcumin and its analogs can efficiently activate TFEB and promote autophagy to therapeutically revive neurodegenerative cells and can be a potential treatment option [68].

In addition to this, Maiti and the team investigated the potential of curcumin to induce autophagy in Aβ_42_ exposed neuroblastoma (N2a), and human cortical neurons (SH‐SY5Y) cells were analyzed. Where, in vitro analysis illustrated an increase in the levels of different molecular chaperones (HSPs), autophagy‐lysosomal pathways (ALPs), and chaperone‐mediated autophagy (CMA) markers. On the flip side, the upregulation of macro‐autophagy markers like LC3A/B‐II and Beclin‐1 was observed in the Aβ_42_ posttreatment group [40]. Jie and co‐workers illustrated the therapeutic potential to treat neural abnormalities using the N2a/APP695swe cell lines. There was a significant increase in the expression of markers including Beclin1, Atg5, and Atg16L1, and promoted expression of dynein, and dynactin leading to the formation of axonal motor complexes to promote fast axonal transport. Also, autophagic induction via co‐localization of both BICD2 and LC3. The formation of dynein, BICD‐2, and dynactin complex modulated the autophagic flux in disease conditions. The curcumin aided in the maturation of autophagosomes and advanced the macroautophagic process in the N2a/APP695swe cells. The author concluded that the curcumin augmented the autophagic flux, and autophagy processes, and could be a potential therapeutic agent to treat neurodegeneration in AD [69]. Jaroonwitchawan et al explored the importance of curcumin in the downregulation of APP, PSI, and PSII in paraquat‐induced SH‐SY5Y cells and the upregulation of antiapoptotic and antioxidant genes. The study reported that there was a decrease in the production of APP and SH‐SY5Y cell protection from apoptosis after curcumin treatment. The suppression of LC3I/II gene expression level was due to curcumin autophagy‐inducing potential [70]. Various other studies suggest the use of curcumin as an autophagy inducer in AD, however, the mechanism is still not fully understood. The observations from various studies suggest the autophagic activity of curcumin in AD, but it exhibits properties associated with pan‐assay interference compounds (PAINS), which can lead to misleading results. These properties include covalent protein binding, metal chelation, redox reactivity, aggregation, membrane disruption, fluorescence interference, and structural decomposition. Studies involving curcumin should therefore be interpreted with caution, particularly in assays that may not fully account for these potential interferences, to avoid overestimating its efficacy in autophagy modulation.

In another investigation, the autophagy augmenting potential of resveratrol was explored in Aβ_25–35_ pretreated PC12 cells by Deng et al. After resveratrol exposure, it remarkably attenuated the detrimental effects of Aβ_25–35_ and revived cell viability. The use of this polyphenol enhanced the formation of autophagosomes, along with a significant decline in a cascade of LC3‐II and p62 degradation. Also, there was an efficiently augmented autophagic revival of neurotoxicity in AD. The possible autophagic induction was through the TyrRS‐PARP1‐SIRT1 signaling pathway. Consequently, Aβ_1‐42_ treated PC12 cells were exposed to resveratrol. They observed elevated acidic vesicular organelles and a boost in Parkin and Beclin‐1 expression, LC3, and TOMM20 co‐localization in the cell lines. Additionally, the resveratrol enhanced mitophagy and promoted the LC3‐II/LC3‐I ratio in the Aβ_1‐42_ treated cells Taken together, the study proved to use polyphenol as a new invader in the therapeutic class of AD [41]. In a similar study, resveratrol‐mediated activation of AMPK and regulation of Aβ peptide metabolism in neuronal and non‐neuronal cells was explored. There was a reduction in levels of Aβ accumulation in primary neurons via AMPK activation. Interestingly, it suppressed the mTOR activity to induce autophagy and caused lysosomal degradation of Aβ. Finally, the activation of AMPK, prevention of Aβ accumulation, and amyloid deposition in the cerebral cortex region in mice in vivo models were observed [71].

Additionally, Choi et al. demonstrated that resveratrol and its analogs like oxy‐resveratrol and piceatannol at higher doses persuaded caspase‐3 and poly ADP ribose polymerase (PARP) cell death, autophagy, and abrogate γ‐secretase activity but piceatannol enhanced α‐secretase activity at a lower dose in Neuro‐2a cells. The study performed in HEK293 expressing APPsw cells suggested a decrease in the level of Aβ in a dose‐dependent manner after the use of these polyphenols without causing cell death. This study further illustrated efficient Alzheimer's treatment and unraveling the likely use of resveratrol and derivatives [45]. Additionally, in different studies, Rahman and colleagues demonstrated AMPK/ULK1/mTOR pathway‐dependent activity of oxy‐resveratrol [44], and Wang et al. showed TFEB/TFE3 mediated augmentation of autophagy by piceatannol in the anticancer immune response of oxaliplatin [72],

Hesperidin, a bioactive flavonoid, was also studied for its autophagic effect against Aβ_25–35_ exposed PC12 cell lines via a VDAC1‐regulated mitochondrial apoptotic pathway. Where the in vitro studies revealed that it prevents the Aβ related apoptosis via Aβ‐induced mitochondrial dysfunction that can lead to disease progression. Also, it demonstrated decreases in ROS production, mPTP inhibition, and suppression in the production of cytochrome *c*, which can lead to the prevention of apoptotic cell death. The authors further extrapolated from the results that the upregulated levels of the VDAC1 phosphorylation can lead to cell death and hesperidin protected by vanquishing mPTP and VDAC1 phosphorylation. Similarly, the activation of the Akt/GSK‐3β pathway subverted the cell viability. In inclusion, the positive effect of hesperidin on PC12 cells unravels the therapeutic use in managing AD [42]. Huang and co‐workers also studied the Aβ_1‐42_ peptide enhanced glucose uptake in insulin‐stimulated Neuro‐2A cells and improved insulin signaling pathways by regulating AKT, GLUT3, and GLUT4 after treatment of hesperetin and hesperidin. From the observations, the authors predicted that these drugs help increase insulin‐stimulated neuronal glucose uptake by inhibiting Aβ‐stimulated autophagy and Aβ‐impaired glucose utilization. In conclusion, they suggested that impairment of energy metabolism leading to neuronal injury can be controlled by the downregulation of autophagy [43].

Leri et. al. have evaluated the effect of extra virgin olive oil polyphenols, namely a mixture of Oleuropein aglycone and hydroxytyrosol in Alzheimer's Disease and have consequently observed reduced levels of autophagy suppressive markers in SH‐SY5Y neuroblastoma cell lines such as ribosomal protein S6 and p62 and elevated levels of phagocytotic markers like Beclin‐1, LC3‐II/LC3‐I ratio [47]. The author concluded that using a polyphenolic mixture (MIX) has significantly shown a preventive effect, however, more profound studies are required such as in animal models to confirm the therapeutic effect. Rigacci and colleagues found out that oleuropein aglycone induces autophagy in CNRND8 mice possessing the APP695 double mutant gene via the AMPK/mTOR pathway [46]. The hydroxytyrosol was explored against Aβ induced toxicity in N2A cells, and the results indicated a protective effect of the polyphenol in the aforementioned model [73].

Furthermore, several other polyphenols have also exhibited autophagy‐inducing characteristics, such as isovitexin [48] and ferulic acid [49]. Isovitexin increased LC3II, Beclin‐1, and (Atg7) expressions in STZ‐injected mice and modulated autophagy via the miR‐107 pathway. In another study, ferulic acid, a common polyphenol, diminished the Aβ levels in *Caenorhabditis elegans* which inhibited Aβ induced paralysis and increased LGG‐1 (autophagy reporter) levels. It was observed that ferulic acid could incite fasting‐like conditions to initiate autophagy in AD, which can be useful in reducing pathological misfolded proteins present in neuronal cells in the disease conditions.

Mitophagy inducers screened via artificially intelligent (AI) tools, namely kaempferol (Kaem) and rhapontigenin (Rhap) were found to restore memory loss [50]. They inhibited both Aβ accumulation, through reduced production as well as phagocytosis, and tau hyperphosphorylation in Aβ_1‐42_ *C. elegans* and 3xTG mice models. Kaempferol enhanced Beclin‐1, LC3B‐II, and AMBRA1 levels and showed a reduction of p62. Rhapontigenin, along with these, also displayed an increase in phosphorylation of ULK1 at Ser555. Additionally, the author revealed that the Kaem can cross the blood‐brain barrier that can be useful in treating neurological manifestations, however, the data for the Rhap is not available.

**Figure 2:**
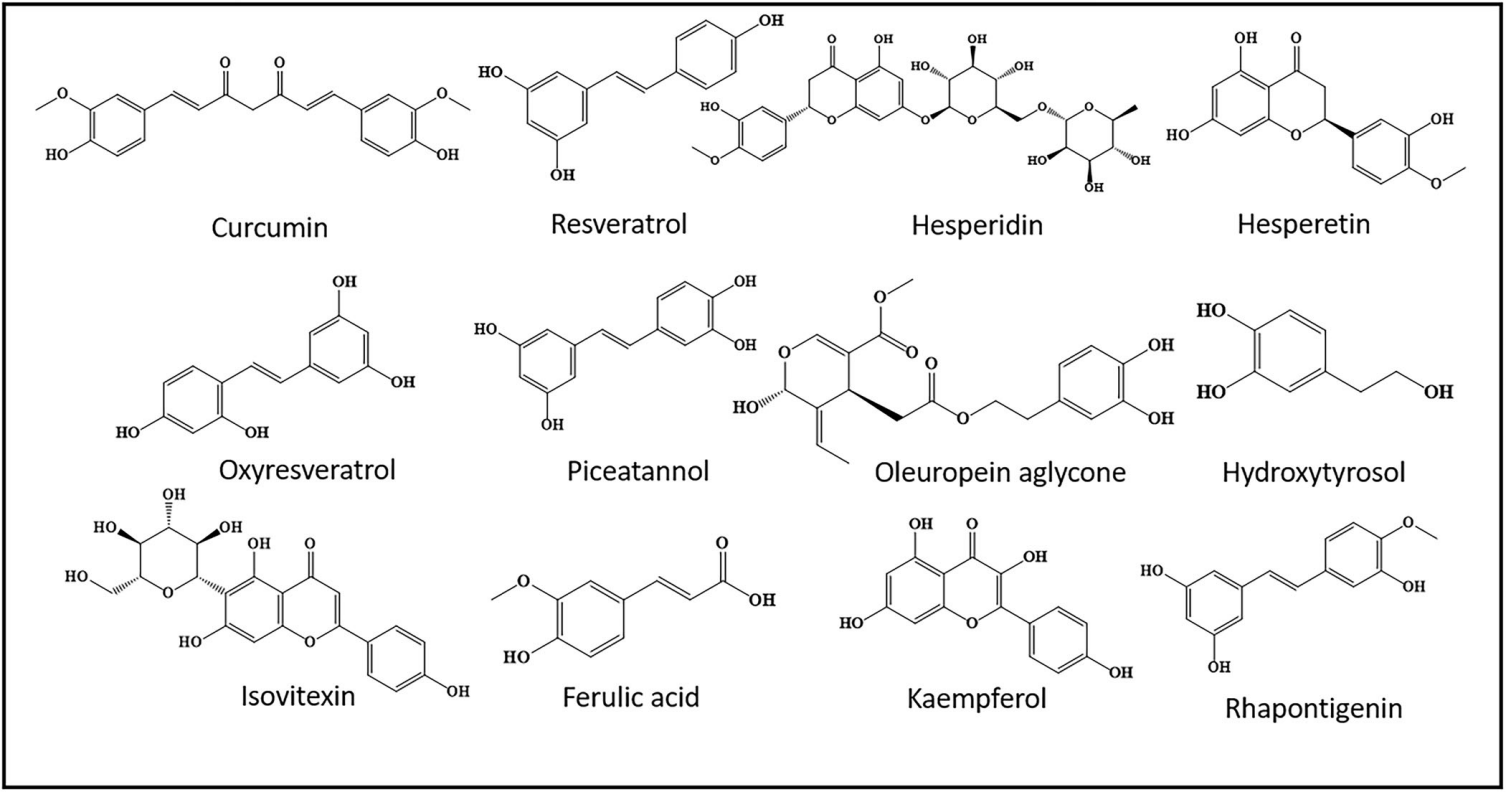
Autophagy‐inducing polyphenolic phytoconstituents.

Harnessing the autophagy‐regulating properties of polyphenols presented in Figure 2 presents a promising avenue for developing interventions that may help mitigate neurodegenerative processes associated with AD. This approach not only highlights the potential of natural compounds in promoting cellular clearance mechanisms but also underscores the importance of exploring novel strategies for AD treatment beyond traditional pharmaceutical approaches.

### 4.2 Alkaloids as Autophagy Inducer

**Figure 3:**
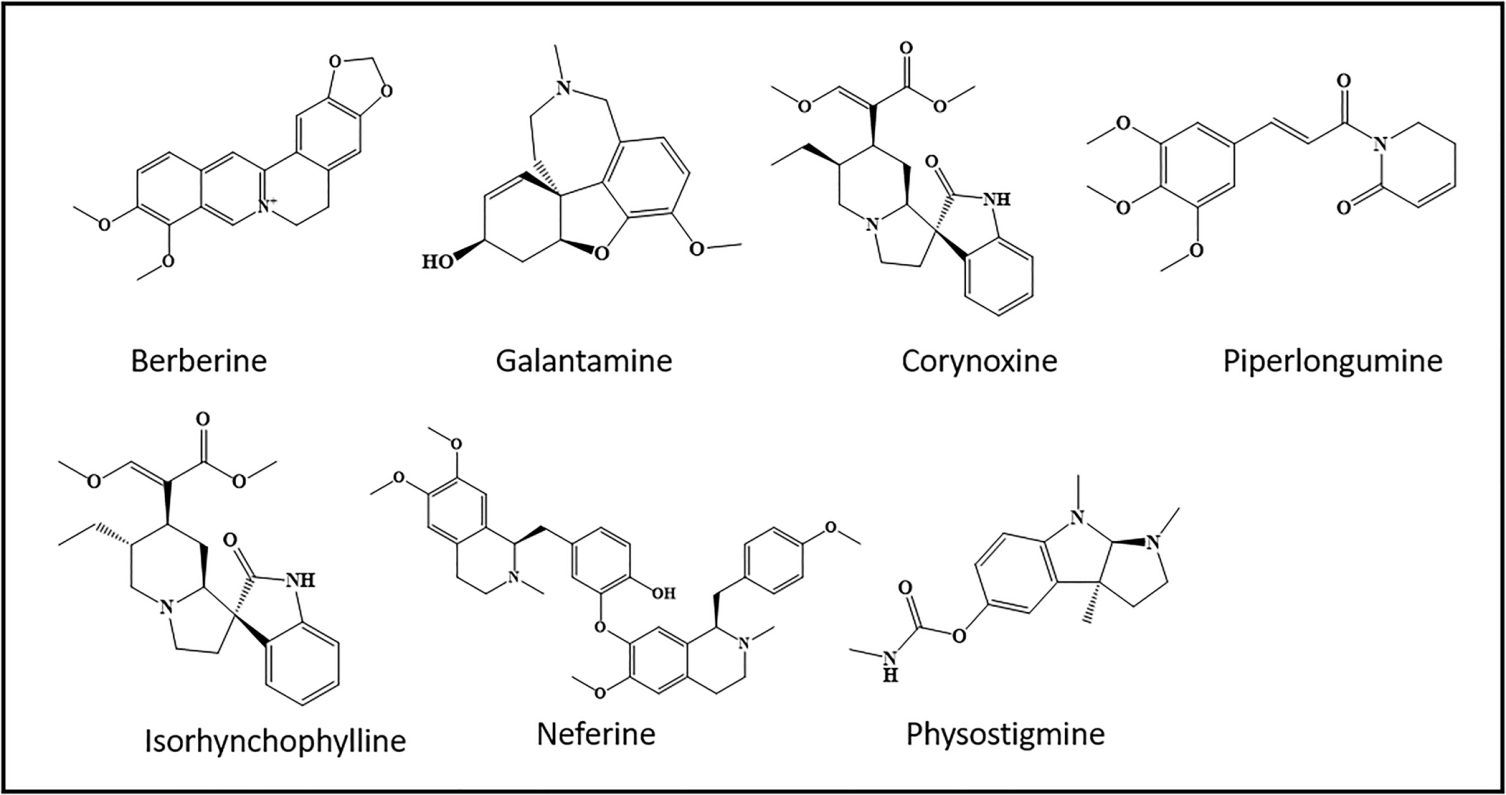
Alkaloidal molecules inducing autophagy.

The alkaloids have also shown autophagic activity in AD models, significantly lowering the disease burden. Various examples are included in this part as well as presented in Figure 3, such as berberine, an ancient alkaloidal ayurvedic medicine obtained from *Berberis vulgaris* is being used in various conditions including Alzheimer's. Huang and co‐workers evaluated the potential of berberine to improve cognitive dysfunction through enhancing autophagic clearance as well as inhibition of ß‐amyloid production in the APP/Tau/PS1 mouse model of Alzheimer's disease [51]. The cognitive functions tests indicated that berberine strongly improves memory functions like learning capacity and memory retention. Additionally, elevated levels of LC3‐II, Beclin‐1, hVps34, and Cathepsin‐D levels as well as the reduction in brain P62 and Bcl‐2 levels in AD mice further confirmed the autophagic induction potential of berberine. In a similar study, the neuroprotective activity of berberine via inhibiting Aβ‐protein‐induced apoptosis through mitochondria‐related caspase pathway in hippocampal neurons primary culture was studied. The cell‐based assays exhibited promising protection against Aβ_25‐35_ protein‐induced apoptosis through the inhibition of caspase‐mediated apoptosis. In addition, Western blot analysis results depicted that change in the expression of cytochrome C, as well as in the levels of Bcl‐2/Bax and Bcl‐xl/Bax ratio in berberine‐treated cells. This further confirmed the neuroprotective potential of this compound against Aβ‐protein‐induced apoptosis [74]. Recently, Chen and team predicted that berberine strongly improves cognitive impairment through tau hyperphosphorylation and autophagic clearance in an experimental model of AD [52]. Behavioral studies have shown that berberine appreciably improves cognitive features and attenuates the hyperphosphorylation of tau through altering Akt/glycogen synthase kinase‐3β and protein phosphatase 2 A activity. The results of Western blot analysis and immunostaining confirmed the reduction in tau level by class III PI3K/beclin‐1 cascade autophagic pathway. Finally, the author summarizes that all the above findings strongly support the strong potential of berberine to treat AD via autophagy. Different studies on the autophagic behavior of berberine concluded its usefulness as a unique compound to treat AD.

Galantamine, a tertiary alkaloid extracted from *Galanthus nivalis* exhibits AChEI activity. It is approved as a cognitive enhancer in AD; however, it is suggested that it has autophagic activity. One such study using galantamine to block the Aβ_1–42_‐induced neurotoxicity through the elevation of the expression of α7nAChR was explored [53]. The cytotoxic evaluation suggests a positive effect against amyloid beta‐induced apoptotic activity via activating the MAPK/JNK and PI3K/Akt pathway inhibition. The results showed increased expression of α7nAChR and inhibition of the Akt pathway via biogenesis of autophagy. The study also indicated expression of α7 subunit protein and binding with autophagosome LC3 protein suggesting the α7nAChRs involvement in the autophagic process. Additionally, the binding of α7 due to the presence of interacting regions for the autophagosome marker protein LC3 was confirmed by immunoprecipitation analysis. The above data indicates the therapeutic potential of galantamine in the treatment of AD.

*Dendrobium nobile* Lindl alkaloid (DNLA) was investigated by Li. and co‐workers for its potential as a novel autophagy inducer and neuroprotective agent by inhibiting Aβ_25‐35_ induced axonal degeneration using hippocampus neuronal cell lines studies. MTT assay and immunofluorescence staining exhibited strong protection by DNLA against axonal degeneration in neuronal cells. A significant overexpression of GFP‐LC3B in DNLA‐treated cells explores its autophagy‐inducing potential and the strong fusion of autophagosomes and lysosomes at desirable pH also suggests its autophagy‐induced caliber. Reduction in PSD95 and SYN protein levels presented novel therapeutic activity as a neuroprotective agent to manage diseases like AD [54].

Corynoxine and isomers were studied as autophagic inducers [55]. The preliminary studies revealed a significant reduction in level resulted in the change in APP levels in Corry and Cory‐B treated cells. Concurrently, a significant increment in an autophagy‐induced marker LC3‐II with Cory and Cory B in a dose‐dependent manner indicated their autophagic activity. The lysosomal degradation abilities of both the compounds were studied using assays like lysosome inhibitor chloroquine (CQ) and autophagy inhibitor 3‐methyladenine, further confirmed by immunocytochemistry of N2aSwedAPP cells using CT15 and LC3 antibody. It was observed that Cory‐B significantly decreases the FI‐APP and APP‐CTF levels in Tg2567. The Cory‐B has a dual action on autophagy and lysosomal degradation, which can be beneficial in AD treatment.

Piperlongumine (PL) is another alkaloid that has exhibited cognitive improvement in numerous studies and has been found to inhibit NFκB and subsequently reduce amyloidogenesis and neuroinflammation in lipopolysaccharide (LPS) administered mice [75]. Also, the levels of TNF‐α, IL‐1β, and IL‐6 were reduced due to a probable link with the NFκB regulation. The data showed a nontoxic effect of PL on normal cells and activation of deacetylase Sirtuin1 (a marker for aging) in neuroinflammation and to enhance cognition in APP/PS1 transgenic mice was investigated [76]. The team also analyzed the amyloidogenic toxicity in HT22 cells and confirmed the significant role of PL in AD. Piperlongumine activates autophagy via enhanced phosphorylation of BCL2 at Ser70, a process that results in its splitting from BECN1 and consequent actuation of autophagy in rotenone‐induced PD mice [56]. This strongly advocates for further exploration of its autophagy‐inducing effect in AD. Interestingly, Isorhynchophylline (IRN) is another alkaloid that through PI3K/Akt signaling alleviates Aβ neurotoxicity via elevation of GSK‐3β synthase and p‐CREB levels in PC12 cells. Although the results from the use of IRN were promising more extensive investigational studies need to be done to prove its clinical use [77]. Various other teams discovered that it also induces BECLIN‐1‐facilitated autophagy in a variety of neuronal cell lines, which has also been found to be independent of the mTOR pathway. This leads to improved clearance of alpha‐synuclein, a protein implicated in the pathogenesis of AD (causes increased tau hyperphosphorylation via GSK‐3β) [57], and Parkinson's disease (PD) [78].

Neferine another alkaloid found in *Nelumbo nucifera Gaerth*, has exhibited potential to diminish Aβ load in APP/PS1 mice, and its inhibitory effect on Aβ was observed by Tht fluorescence technique. In a recent study, exosomes of reference fabricated by high‐speed centrifugation showed reduced levels of p62 and LC3, which validated its inducing effect on autophagy. It also enhanced the conversion of LC3‐I to LC3‐II in PC12 cells, evaluated via quantification of GFPLC3‐II, and increased autophagosome formation. The findings confirmed the reduction in HTT74, P301L tau, and A53T α‐synuclein levels after exosome application via autophagy in the cell line model [58].

Physostigmine was examined by Haefen and colleagues for its potential to restore the autophagy in the rat hippocampus model impaired post‐surgery stress and LPS Treatment [59]. *In vitro* studies showed a significant increase in apoptosis, autophagy, and anti‐inflammatory‐related proteins in physostigmine‐mediated hippocampal cells—moreover, physostigmine treatment results in prominent attenuation of expression of autophagy‐induced p62/SQSTM1 marker post‐surgery stress. The reduction in expression of apoptotic‐related proteins such as TGF‐beta1 and MFG‐E8 after surgery stress was confirmed via fluorescent examination. Hence, physostigmine has strong neuroprotective potential through autophagy‐inducing capability against surgery‐induced stress as well as inhibition of cognitive dysfunction.

Additionally, the potential therapeutic effect of caffeine in inhibiting the HIV‐1 Tat‐induced Aβ production and tau phosphorylation. The exploratory studies in human neuroblastoma cells (SH‐SY5Y) using ELISA assay revealed a prominent decrease in Aβ level and significantly blocked HIV‐1 Tat‐induced Aβ generation in caffeine‐treated cells. The immunoblotting displayed that the caffeine prominently decreases the vascular‐ATPase levels and increases cathepsin D levels via HIV‐1 Tat‐induced endo‐lysosome dysfunction. Based on the above findings author summarizes that caffeine might be used as an effective therapeutic candidate in combination with other drugs to treat AD [79].

### 4.3 Glycosides as Autophagy Inducer

**Figure 4:**
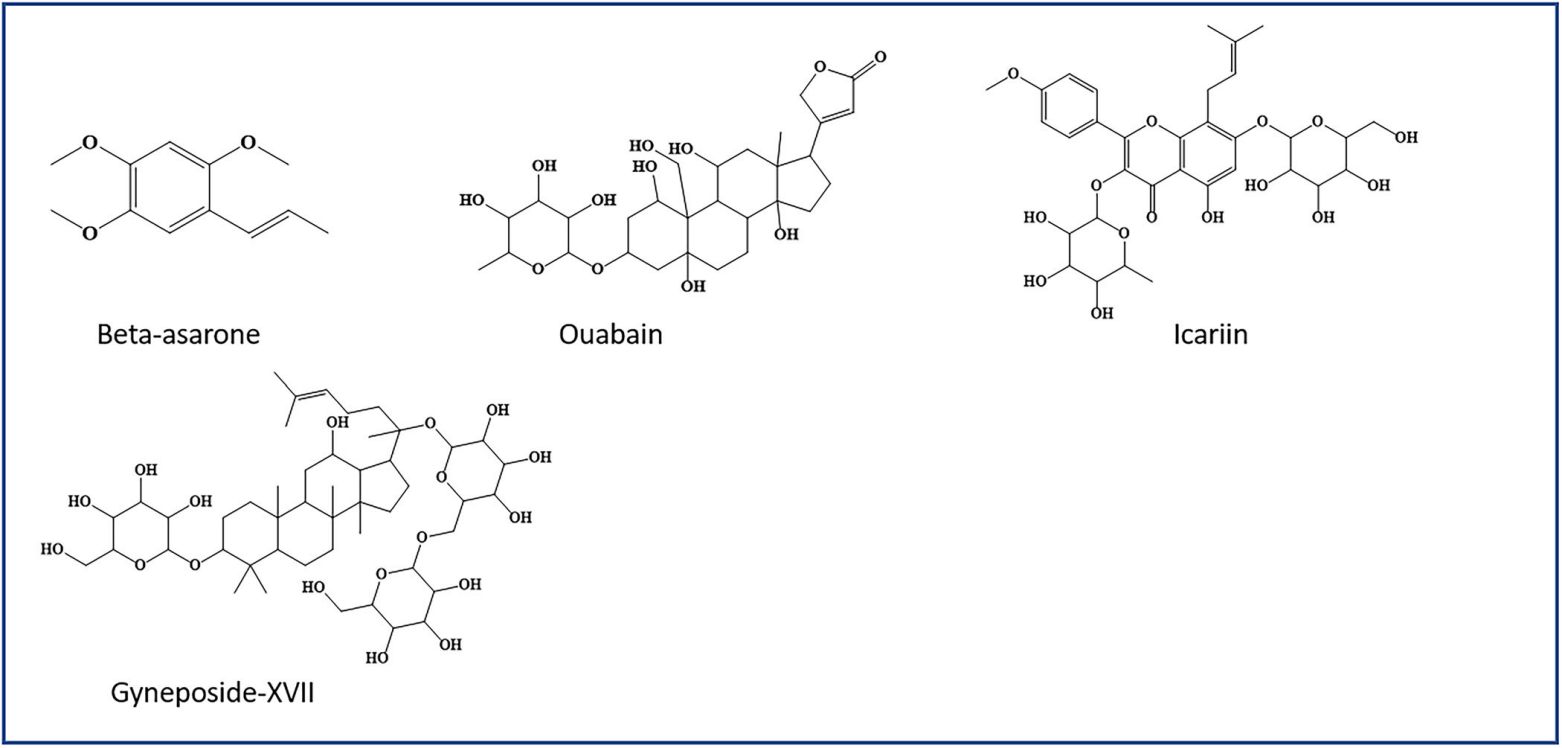
Autophagy inducing glycosides. [Color figure can be viewed at wileyonlinelibrary.com]

Glycosides are naturally occurring compounds and have a glucose moiety attached to the glycogen part with a glycosidic bond. From ancient times their wide pharmacological activity in different diseases has been proven. Similarly, the role of different glycosides as autophagy inducers is summarized in this section and Figure 4.

In a such study, Grossi and team evaluated the potential of polyphenol oleuropein aglycone to ameliorate Aβ plaque deposition using the TgCRND8 mice model. They showed that with oleuropein aglycone treatment, a significant reduction in the levels of beta‐amyloid and plaques through phagocytosis along with the decrease of the astrocyte's reaction. The authors further suggested that there was a strong autophagic reaction in oleuropein aglycone‐fed mice brains. Based on the above finding, it was suggested that using these compounds as a dietary supplementation can be beneficial for AD treatment [80].

The potential of *Ginkgo biloba* (EGb 761) extracts to improve the symptoms and pathology of Alzheimer's in a transgenic mouse model. The behavioral studies using maze tests indicated significant changes in the cognitive activities of mice. A promising inhibition of inflammation along with Aβ protein aggregation and enhancement in autophagy biomarkers was seen in EGb 761 treated mice. The authors suggested that prolonged usage of EGb 761 induces autophagy and improves both behavioral and cognitive functions in AD models [81]. In a recent study, the anti‐Alzheimer potential of combined hyperbaric Oxygen and *Ginkgo biloba* Extract by inhibiting Aβ_25‐35_‐induced toxicity and oxidative stress [82]. The authors demonstrated a significant improvement in cognitive function via activation of SOD after treatment and a significant reduction in malondialdehyde (MDA) levels but negligible change in nitric oxide (NO) productions. Hence, the combination treatments have a strong neuroprotective potential and a protective role against AD.

The neuroprotective effect of β‐asarone via increasing expression of SYP and GluR1 in both APP/PS1 2x transgenic mouse model and NG108 cell lines was explored. The findings of cell studies revealed insignificant toxicity as well as change in cell morphology after β‐asarone treatment. The western blot analysis illustrated significant elevation in the expression of SYP and GluR1 in dose dose‐dependent manner. Finally, the in vivo, and in vitro data demonstrated the neuroprotective potential of β‐asarone is sufficient to consider this herbal drug for AD management [83].

Song et al explored the neuroprotective potential of Ouabain (OA) via activating transcription factor EB in Alzheimer's disease models. The two‐step screening tests showed OA‐induced neuroprotection and overexpressed TFEB‐medicated protection against neuronal damage. Also, the autophagy‐enhancing caliber of Ouabain was confirmed in the treatment group. Additionally, the expression of the macro autophagy gene elaborated a significant reduction in tau phosphorylation in okadaic acid‐neurodegenerative models. So, based on the above findings authors concluded that autophagy activation using OA can lead to the development of practical treatment for neurodegenerative diseases like Alzheimer's [60].

Yang and co‐workers examined the cornel iridoid glycoside potential to induce autophagy, where the glycogen synthase kinase‑3β activated protection in the tau neurotoxicity was tested. The cognitive tests exhibited significant improvement in memory dysfunction after oral administration. Increased expression of presynaptic *p*‐synapsin, synaptophysin, and postsynaptic density‐95 can be related to the reduced levels of tau oligomer in the brain of WM/GFX rats and cell lines. Furthermore, the expression of markers like ATG7, ATG12, Beclin‐1, and LC3II was seen in both in vivo and in vitro, which confirmed the autophagic process. Based on the above data it can be suggested that cornel iridoid glycoside has the potential to restore autophagy and treat various illnesses [61].

The biological activity of DMDD isolated from the roots of *Averrhoa Carambola L*. to protect against neuronal damage and memory impairment was studied by Wei et al. The Y‐type electric maze behavioral study illustrated a significant improvement in learning and memory functions. A prominent reduction in neuronal damage and apoptosis in DMD‐treated cells using Annexin‐V and histopathology was seen. Further supported by a significantly improved ratio of Bcl‐2/Bax in DMDD‐pretreated in vitro cell lines and in vivo mice models. In a nutshell, it can be recommended that DMDD usage can be beneficial against AD [62]. In another study, the neuroprotective role of Icariin for improved synaptic and cognitive deficits in an A*β*_1–42_‐induced experimental model of AD was studied [63]. Behavioral studies depicted that there was a significant of learning and memory in different ICA‐pretreated and untreated groups. The immunohistochemical assay gave a dose‐dependent (at low to high) prominent increase in the immuno‐positive cells in the hippocampus region. Also, the Western blot analysis and PCR investigations showed decreased levels of various biomarkers. The findings supported the autophagy‐induced improvement in synaptic plasticity through the BDNF/TrkB/Akt pathway, where implying its clinical application in AD.

Meng and co‐workers evaluated Gypenoside XVII (GP‐17) potential to enhance lysosome biogenesis and accelerate autophagic clearance through TFEB activation in cell lines of APP695 (APP695swe) and APP/PS1 mice. The cell‐based assays showed an increased Atg5 expression and a reduction in p62 expression in GP‐17 treated cells. The autophagy‐promoting potential of GP‐17 was also explored through the elimination of amyloid proteins in the cells preventing the segregation of Aβ plaques. The memory functions test demonstrated an increase in learning and memory function. The Western blot analysis confirmed the enhanced staining and amplified LAMP‐1/LC3‐II co‐localization. Authors suggested that GP‐17 could release TFEB from TFEB/14‐3‐3 complexes and these translocations could result in lysosomal biogenesis and autophagy. It was established from the results that it exhibits conferred protective effects in the Alzheimer's model [64].

### 4.4 Miscellaneous Phytoconstituent Compounds as Autophagy Inducers

**Figure 5:**
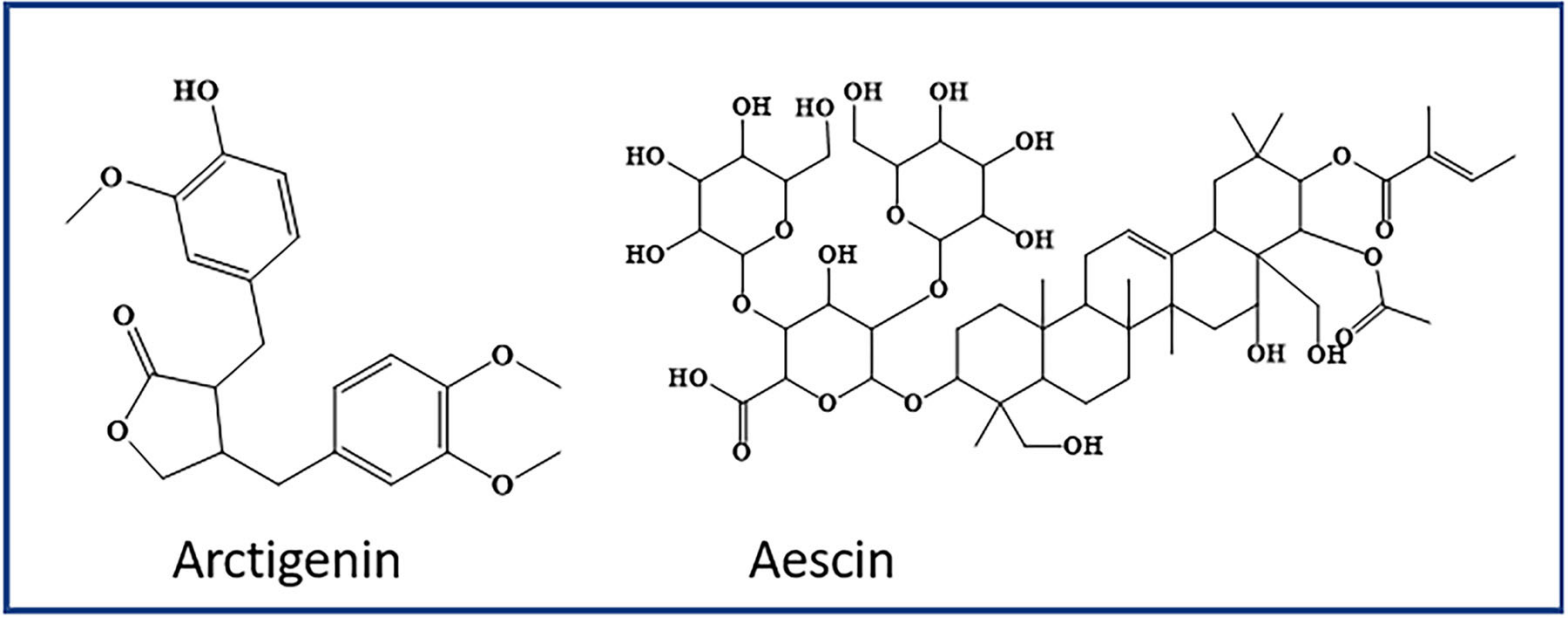
Autophagy‐inducing miscellaneous compounds. [Color figure can be viewed at wileyonlinelibrary.com]

Besides these polyphenols, alkaloids, and glycosides, some other miscellaneous natural compounds from different classes show autophagic activity. These miscellaneous compounds are presented with their structures in Figure 5. Claims from various studies that are briefly summarized, such as Zhu et al. aim to assess the improved cognitive activity of arctigenin in an AD model. This study illustrated that it acts on APP enzyme and suppresses amyloid beta production. Also, in the APP/PS1 mice model, it was observed that the plaque clusters were cleared through autophagic induction via inhibiting the AKT/mTOR signaling pathway. In addition to this, inhibition of the PERK/eIF2α pathway declines the translation of BACE1 and finally reduces the production of plaques in disease conditions. The findings from the study sufficiently claim that arctigenin activity enhances memory and cognitive function in the disease model [65].

The functional mechanism of β‐asarone, belonging to the phenylpropanoid class, on autophagy in Aβ induced cell injury is not understood yet and there is not much information on the autophagy regulation pathway of β‐asarone. Therefore, Xue and team explored the β‐asarone anti‐amyloid cytotoxicity through BECN1 complex‐dependent autophagy in the cells. They found that β‐asarone sustained the cell's original morphology, enhanced the cell's viability, and lowered the NSE levels. Moreover, β‐asarone lowered the Beclin‐1 expression and enhanced AKT and mTOR levels. The study depicted that asarone exhibits its anti‐amyloid activity by promoting autophagy AKT‐mTOR pathway [84]. In many of the cells, sulforaphane (SNF) has been described as an autophagy inducer by activating the nuclear factor E2‐related factor 2 (Nrf2‐E2). Therefore, Jo et al aimed to study autophagy inducing potential of SNF using LC3‐II levels. They reported that SFN could act as an autophagy inducer by activating the ERK and independent of Nrf2‐E2 activity in neuronal cells [66].

Escins are saponins with vasoprotective, neuroprotective, and anti‐inflammatory effects. The role of escins present in *Aesculus chinensis Bge*. in autophagy induction has been explored in HT22 cells and identified to be mediated via mTOR and ERK signaling. They were found to inhibit the PI3K‐AKT pathway and promote AMPK signaling, in consequence leading to the inhibition of mTOR signaling. Moreover, they also caused the downregulation of P70S6K and ULK1 as well as a reduction in LC3‐II and p62 levels. In conclusion, these molecules showed protective effects in neurological manifestations including AD [67].

## 5 Memantine: An Approved Drug as Autophagy Inducer

Memantine is an FDA‐approved drug for AD. Its mechanism of action is commonly explained via its noncompetitive NMDA receptor‐blocking activity, which prevents the influx of excess Ca^2+^ ions into the neuronal cells, and consequently prevents neuronal damage and apoptosis. It also increases Aβ_42_ clearance, but autophagy has also been suggested as a main mechanism [85]. Hirano and colleagues observed the increase in LC3‐II levels in the HeLa cell line by memantine and also concluded that the autophagic effects of memantine did not show involvement of mTORC1 and NMDAR pathways but may alter the activity of the VPS34 complex. They harvested Induced pluripotent stem cells (iPSCs) from the dermal fibroblast of a healthy subject, a PARK2 and a PARK 6 patient, and measured differentiation efficiency as well as mitophagic flux. Where they observed autophagic flux in damaged mitochondria of both PARK2 and PARK6 patient neurons via PINK1/parkin signaling.

Another group of researchers, Wet and colleagues, observed the concentration‐based effect of memantine on mitochondrial networks, with dose‐dependent macro‐autophagy and mitophagy activation. Júlia Companys‑Alemany et al illustrated the memantine‐autophagy relation in 5XFAD mice, where it diminished the Aβ levels and improved cognitive function. The induction of autophagy was indicated by elevated levels of autophagy markers such as LC3‐II/LC3‐I ratio, and LAMP1, whereas no alteration was seen in p62 levels [86]. In similar works, it was demonstrated that memantine can exhibit autophagy‐inducing capabilities in other disease models. One such research by Wan‐Soo Yoon et al. illustrated its effect on malignant glioma cell lines (T‐98 G and U‐251 MG) and discovered autophagy‐induced cell death. An increase in LC3‐II/LC3‐I ratio and Beclin‐1 levels was observed along with enhanced formation of autophagic vacuoles via TEM imaging. Also, the role of NMDR1 receptors was confirmed in autophagy in T‐98 G cell lines [87]. Along similar lines, Albayrak et al. explored the effects of memantine on lung cancer lines (A549) and estimated via the Western Blot technique, the levels of LC3B‐I, LC3B‐II, and SQSTM1 proteins. There was an increase in the expression of LC3B‐II and SQSTM1 after treatment with memantine, indicating a prominent role in autophagy [88]. However, the studies have shown G0/G1 cell cycle arrest in cancer cells, but its autophagic role in different disease conditions needs to be explored.

In 2019, a novel H_2_S‐releasing prodrug of memantine was synthesized by Sestito and group, by substitution of free NH_2_ with H_2_S, thereby equipping the molecule with H_2_S‐releasing property. Memantine and the newly formed compound, named ‘mention’, upregulated LC3II expression, while reducing expression of p62, p‐mTOR proteins as well as Akt‐Ser473 phosphorylation. This indicated that both of these molecule's function via modulation of PI3K/AKT/mTOR signaling. Memantine and memit inhibited self‐aggregation of Aβ_1‐42_ as well. In conclusion, the author believes that besides its disease‐modifying potential and autophagy inducer, its use is still limited as an NMDA receptor blocker in neurodegenerative conditions [89].

## 6 Drug Repurposing in Autophagy

Extensive research has gone into drug development and delivery, which has subsequentially led to promising therapeutic APIs being approved for various pathologies by various regulatory agencies such as the Food and Drug Administration (FDA). These APIs have been approved only after much deliberation and toxicological and pharmaceutical evaluation. This availability of details has led to the exploration of the use of these drugs approved for a certain disease for other pathologies, prominent examples being drugs such as minoxidil (alopecia areata), sildenafil (erectile dysfunction), aspirin (angina), and so forth. Also, there is incessant research into medications and drugs for conditions that have unmet needs or have only symptomatic treatment options available, for example, ulcerative colitis, rheumatoid arthritis, various carcinomas, PD, AD, and so forth.

Certain FDA‐approved drugs have been found to induce autophagy in various studies. Also, some of these have good blood bioavailability, Aβ accumulation inhibition, and tau hyperphosphorylation blocking capabilities to name a few, which have also been demonstrated by various scientific groups listed in Table 2.

**Table 2:** Repurposed molecules with Autophagic potential via different AD management pathways.

Drug	Approved use	Mechanism	Pathway	Significance	Reference
Carbamazepine	Antiepileptic	Na^+^/Ca^++^ channel inhibitor	AMPK activation	CBZ reduces Aβ deposition and Aβ levels by triggering the AMPK pathway	[90, 91, 92, 93, 94]
Rapamycin	Organ transplant rejection	mTOR inhibitor	mTOR inhibition	mTOR inhibition‐dependent inhibition leads to reduced Aβ levels and tau hyperphosphorylation	[95, 96, 97]
Everolimus	Anticancer	mTOR inhibitor	mTOR inhibition	mTOR inhibition‐dependent inhibition leads to reduced Aβ levels and tau hyperphosphorylation	[98]
Temsirolimus	Anticancer	mTOR inhibitor	mTOR inhibition	mTOR inhibition induces autophagy and reduces Aβ levels while reducing cognitive decline, but low bioavailability an issue	[99, 100]
Bosutinib	Anticancer	c‐Abl inhibitors	beclin‐1 and parkin‐mediated induction	Tyrosine kinase inhibitor with autophagy inducing property in AD preclinical models	[101, 102, 103, 104]
Nilotinib	Anticancer	c‐Abl inhibitors	AMPK activation, HDAC inhibition	Induction of autophagy to lower Aβ levels in preclinical models	[105, 106]
Dasatinib	Anticancer	c‐Abl inhibitors	Beclin 1, AKT, and Bcl‐2 induced	A hydrophobic anticancer drug with Beclin 1, AKT, and Bcl‐2 mediated autophagy inducing property	[106, 107, 108]
Dapagliflozin	Type‐2 diabetes	SGLT2 inhibitor	LKB1/AMPK/SIRT1/mTOR pathway	An antidiabetic SGLT2 inhibitor with LKB1/AMPK/SIRT1/mTOR mediated autophagy induction	[109, 110, 111, 112]
Liraglutide	Type‐2 diabetes	GLP‐1 analog	AMPK/mTOR pathway	Peptide‐based drug which activates autophagy via the AMPK/mTOR pathway, low patient compliance is a major concern	[113, 114, 115, 116, 117]
Semaglutide	Type‐2 diabetes	GLP‐1 analog	↑LC3II, Atg7, Beclin‐1, and P62 levels	Peptide drug which induces autophagy by increasing ↑LC3II, Atg7, Beclin‐1, and P62 levels, but with low oral bioavailability	[118, 119, 120]
Metformin	Type‐2 diabetes	Antihyperglycemic agent	Chaperone‐mediated autophagy	An antidiabetic drug that induces autophagy via multiple reported pathways	[121, 122, 123, 124]
Simvastatin	Anti‐hyperlipidemic	HMG‐CoA reductase inhibitor	mTOR inhibition	A statin with mTOR inhibition‐mediated autophagy induction, however, efficacy is doubtful due to no improvement noted in clinical trials, further research needed	[125, 126, 127]
Gemfibrozil	Anti‐hyperlipidemic	PPARα agonist	PPARα mediated autophagy	PPARα agonist induces the PPARα pathway which triggers autophagy	[128, 129, 130]
Nicotinamide	Pallegra	Micronutrient	mTOR, AMPK, mTORC1, and mTORC2 mediated autophagy	HDAC inhibitor which induces autophagy via multiple pathways, the high dose required for therapeutic effect in AD causes hot flashes and hepatotoxicity	[131, 132, 133, 134, 135]
Vorinostat	anticancer	HDAC inhibitor	PIK3C3/VPS34‐BECN1 mediated mitophagy	Anticancer drug which induces autophagy via PIK3C3/VPS34‐BECN1 mediated pathway	[136, 137]

These preclinical studies render them suitable candidates for use as autophagy inducers in AD. Here, we will briefly review some of the FDA‐approved drugs, focusing on their role in AD and autophagy modulation.

Carbamazepine (Dibenzoazepine): Carbamazepine (CBZ) is an FDA‐approved anticonvulsant, that has been explored in the induction of autophagy in AD by Lixi Li and colleagues [91]. They observed a notable reduction in Aβ plaque load in APP (swe)/PS1(deltaE9) mice after treatment with CBZ. This was accompanied by the induction of autophagy and reduction in mTOR signaling, both of which were notably independent of each other. Further enlightenment over this was provided through a study by Zhang et al., who also found the autophagy induction of CBZ to be independent of the mTOR pathway, along with an increase in LC3‐II expression and diminished Aβ_42_ levels. However, dose‐dependent toxicity was also observed [90].

Several clinical studies have focused on the calming effect of CBZ on AD‐related agitation. A couple of clinical studies found CBZ to be efficacious in treating AD‐linked agitation [92, 93]. Certain drug‐related side effects were also noted such as agranulocytosis, leukopenia, and allergic reaction. However, a recent trial, namely the SYMBAD trial (NCT03031184) reported that neither CBZ nor another antidepressant, mirtazapine, could alleviate or diminish agitation related to AD, while duly mentioning that the CBZ group was discontinued due to low recruitment [138]

Rapamycin, everolimus, and temsirolimus (Macrolides): Also called sirolimus, rapamycin is an mTOR inhibitor and a known autophagy inducer. It is an FDA‐approved medication having multiple FDA approvals under its belt, viz kidney transplant rejection (1999), restenosis (2003), locally advanced metastatic or unresectable malignant perivascular epithelioid tumors (2021) and Lymphangioleiomyomatosis (2022) [95]. Rapamycin has exhibited anti‐AD activity in multiple animal models, such as APP/PS1 and P301S mice, leading to augmented memory function and attenuated Aβ load and tau hyperphosphorylation [96]. Currently there are two ongoing clinical trials for evaluating the safety and efficacy of rapamycin in AD, the Evaluating Rapamycin Treatment in Alzheimer's Disease using positron emission tomography (ERAP) phase IIa trial and another phase II trial (NCT04629495) for evaluation of safety and efficacy of rapamycin in early‐stage AD [139].

Temsirolimus is an ester of rapamycin and a 2nd gen mTOR blocker that has been FDA‐approved for renal cell carcinoma. This drug in its complex form with FKBP12 binds to mTOR at its piperidine region causing mTOR to dimerize and inactivate [99]. Another mTOR inhibitor, namely everolimus has emerged as a treatment option for early stage dementia, courtesy of a study on its effects on a 3xTG‐AD mice model. It was administered via an osmotic pump into the brain to overcome its low BBB crossing capability. A higher half‐life in the brain was recorded leading to slower metabolism. A notable enhancement in cognition and a lower negative impact on the periphery were seen, suggesting that intrathecal administration of the drug may improve neurodegeneration via inhibition of mTOR [98]. Another study by Jiang and colleagues on HEK293‐APP695 cells and APP/PS1 mice validated the anti‐AD activity of temsirolimus, which marked a reduction of hippocampal apoptosis and Aβ plaques, as well as improvement in memory and cognition [140].

Bosutinib, nilotinib, dasatinib (c‐Abl inhibitors), and masitinib (Tyrosine kinase inhibitor) (Benzamides): These benzamides have been extensively used in the treatment of leukemia and are FDA‐approved. Research by Irina Lonskaya and colleagues has unraveled that nilotinib and bosutinib increase the solubility of parkin and lead to enhanced parkin‐beclin‐1 interaction, which subsequentially leads to increased Aβ clearance and reduction in plaque load [101, 141]. A clinical study by Pagan et al. explored the effects of bosutinib in dementia with Lewy bodies (DLB) patients and noted that the drug was safe and well tolerated in DLB patients, with attenuation of alpha‐synuclein and dopamine levels in CSF, at approximately the least efficacious dose of bosutinib [103]. In another study by Mahdavi et al., it was observed that bosutinib when administered in patients with probable Alzheimer's dementia or Parkinson's spectrum disorder with dementia responded positively to the therapy over 1 year, and the cognitive and behavioral symptoms worsened with discontinuation of the therapy (NCT02921477) [102]. This study was further followed up 1 year post earlier publication with findings along similar lines, further warranting the continuation of studies investigating the therapeutic role of bosutinib in dementia and AD [142].

Dasatinib is also a promising TKI explored by multiple groups for its effect on cognition [143, 144] undergoing clinical trials in combination with quercetin. Adam Krzystyniak and colleagues evaluated the dasatinib and quercetin combination for efficacy in AD in senescent Wistar rat models. They noticed a significant improvement in cognitive ability as well as spatial awareness using active allothetic place avoidance test, also they confirmed that the combination therapy restored hippocampal functions [145]. Both quercetin (as discussed above) and dasatinib have autophagy‐inducing activity. There have been multiple reports of autophagy‐inducing activity of dasatinib in a wide array of disease models, viz ovarian cancer [107], leukemia [146], and malignant pleural mesothelioma [147]. In brief, it enhanced beclin‐1 expression, p62 degradation, and LC3B‐II levels and reduced AKT expression. The clinical investigations of the combination therapy have been discussed in a later section containing drugs in clinical trials for AD with linked autophagy mechanisms.

Dapagliflozin (c‐glucoside Sodium‐glucose co‐transporter‐2) got FDA approval for disease conditions like heart failure and type‐2 diabetes, where it blocks the reabsorption of glucose from proximal convoluted tubule. Its role in preventing heart failure is under scrutiny, however, the potential mechanism is through diminishing calcium levels in cardiac myocytes and prevention of cardiac remodeling [148]. In addition to this, Furuya et al. explored the autophagic potential of dapagliflozin in the liver and found that the prominent hepatic autophagy, where the rise in LC3‐II: LC3‐I ratio along with inhibition of mTORC1 was observed through SGLT2 inhibition. However, the expression of p62 remained unaltered [109]. In a similar study by Li et al., it was observed that the inhibition in AMPK‐mTOR signaling (enhanced activation of AMPK and decreased dephosphorylation of mTOR) and thus induces autophagy in hepatic steatosis‐induced HepG2 cells and ZDF rat models [110]. The same phenomenon was further investigated in cadmium‐induced testicular dysfunction in rat animal models. The upregulation of Beclin 1 and a decrease in SQSTM‐1/p62 accumulation lead to the activation of the autophagic pathway [149]. Samman et al examined the role of dapagliflozin therapy in aluminum chloride‐induced dementia in a Swiss rat model, which was further investigated by behavioral as well as mechanistic studies. The results from the treatment group exhibited a lower mean escape latency and improved target recognition in the Morris Water Maze (MWM) test. Also, the rise in total entries as well as spontaneous alternations in the Y‐Maze test were seen. The higher p‐AMPK and p‐mTOR activation resulting in reduced oxidative stress and inflammatory markers were further supplemented through western blot analysis. In conclusion, dapagliflozin showed a neuroprotective effect in the induced AD model, but further investigation is needed to unravel the full potential [111]. A phase‐2 clinical trial (NCT03801642) exploring the safety, tolerability and efficacy of 10 mg dapagliflozin administered in probable AD patients was performed, with results not disclosed till present [150].

Liraglutide and Semaglutide (Glucagon‐like peptide (GLP‐1) analog): Liraglutide got its approval for type 2 diabetes in pediatric patients and weight management drug in children of 12 and above (fda.gov, 2019,2020). Liraglutide has been reported to increase LC3‐II, beclin‐1 levels, and Atg7 levels in high‐fat diet rats and HepG2 cell lines. It also enhanced the activation of AMPK and reduced p‐mTOR expression. The authors concluded that liraglutide induced autophagy via the AMPK/mTOR pathway [113]. Another study explored the effects of liraglutide on hepatic steatosis. It was observed that the lipopeptide increased autophagic flux via elevated nuclear translocation of Transcription factor EB (TFEB) and Increased CTSB and LAMP1 expression which are involved in downstream signaling in the TFEB pathway [114]. The autophagic induction by liraglutide was further validated by Yuan and colleagues, in an erectile dysfunction rat model using cavernous nerve electrostimulation. The study revealed that the drug alleviated erectile dysfunction via GLP‐1R expression leading to autophagy along with reduced ROS levels [115]. Moreover, its role in the treatment of AD was further assessed in the phase IIb clinical trial (NCT01843075), where it was assessed as a GLP‐1 analog and showed a notable improvement in cognitive capabilities and MRI volume [116]. Liraglutide has also been explored as a dual treatment option for type 2 diabetes and AD in APP/PS1xdb/db mice. The results indicated that treatment with liraglutide may prevent brain damage in Type 2 diabetes when AD is concurrent but clinical application is a bit debatable [151]. However, McClean and colleagues have thoroughly explored the anti‐AD effect of liraglutide in various prophylactic and AD‐induced APP/PS1 mice models and validated the therapeutic efficacy of this GLP‐1 analog. Liraglutide exhibited the potential to reduce cognitive decline in mild AD patients by up to 18% when compared to the placebo group due to a decrease in the rate of shrinkage of cognitive parts of the brain [152].

Semaglutide is a Glucagon‐Like Peptide‐1 receptor agonist approved by the FDA for weight loss and type‐2 diabetes in adults [153]. In a similar study, it also induces autophagy in SH‐SY5Y cells impaired by Aβ_25‐35_. This was validated by an increase in Atg7, Beclin‐1, p62, and LC3‐II levels in semaglutide‐treated cell lines [118]. Another study reported that semaglutide may have a positive role in diminishing dementia in patients with type‐2 diabetes [154]. Currently, two phase‐3 clinical trials, EVOKE (NCT04777396) and EVOKE+ (NCT04777409) are being conducted to garner information related to the safety and efficacy of this autophagy inducer in early‐onset AD [155].

Metformin (Biguanide): Metformin is the drug of choice for type‐2 diabetes. It has a very good safety profile and therapeutic window [156]. To validate the autophagic flux enhancement by metformin in cancer pathogenesis, Mauro de Santi and colleagues performed extensive study on multiple cell lines, viz, NIH/3T3, 3T3‐619C3, 3T3‐619D3, 3T3‐621B1, 3T3‐621B6, and3T3‐SCRD3 cell lines previously treated with MNU and H_2_O_2_ (for induction of tumorigenesis) and observed cytostasis in all of these, indicating an autophagic effect on cancer cells. This was further confirmed by an increase in LC3‐II and p62 expression. Also, NIH/3T3 and 3T3‐SCRD3 cell lines exhibited reduced cell viability, whereas shATG7 cell lines did not show the same, indicating that metformin‐induced autophagy in impaired cells [157]. There have been multiple reports of autophagy induction due to metformin treatment through a wide spectrum of pathways [158]. Metformin has been reported to enhance AMPK activation in the cerebrum and decrease mTOR and NFκB in APP/PS1 mice [121]. Furthermore, Campbell and colleagues discovered via a meta‐analysis of clinical studies, that metformin administration leads to reduced risk of dementia in patients with type‐2 diabetes [159]. However, metformin may cause vitamin B12 deficiency which has been reported to have a significant role in memory functions and cognitive ability [160, 161]. A case‐control study by Janet et al. investigated the risk of AD in diabetes patients administered with metformin. The team concluded that metformin not only does have any AD‐related risk, but contrarily it was associated with a lower risk of AD incidences in older diabetic patients [123]. A pilot clinical study exploring the safety and tolerability of metformin in Luchsinger et al. in prevention and treatment of hyperinsulinemia in patients with amnestic mild cognitive impairment (AMCI). The study revealed that metformin moderately improved cognitive symptoms while not causing any serious adverse effects. Metformin was tolerable at different doses for different patients, where only 10% of patients were able to tolerate the maximum dose of 1000 mg twice a day [122].

Simvastatin (hexahydro naphthalene): Statins are antihyperlipidemic drugs that inhibit HMG‐CoA reductase enzyme leading to reduced cholesterol levels, and therefore find their use as an adjuvant in a plethora of cardiovascular diseases as well, such as hypertension, angina, etc [162]. Simvastatin is derived from lovastatin which was the first statin to get FDA approval. Simvastatin is FDA‐approved as an antihyperlipidemic, however recent research has explored this statin for its therapeutic efficacy in a wide array of diseases, such as atherosclerosis [163], osteoarthritis [125], alcohol‐induced liver disease [164], hepatitis C [165], hepatocellular carcinoma [166], etc. with improvement in aforementioned pathologies via induction of autophagy. Wei and colleagues studied the effects of simvastatin in coronary artery myocytes (CAMs) and discovered that the statin significantly enhanced LC3B and Beclin1 levels. Moreover, the autophagy induction was confirmed by flow cytometry by which it was found that autophagosome generation was increased, and by confocal microscopy which permitted them to visualize the elevated lysosomal fusion of the autophagosomes, which reduced with the introduction of 3‐methyladenine, an autophagy blocker, or Atg7 gene knockdown. The mTOR‐dependent autophagy induction of simvastatin‐conjugated gelatin hydrogel administration in 10‐week‐old osteoarthritis‐induced C57BL6/J mice was demonstrated by Tanaka et al. The hydrogel reduced mTOR phosphorylation and IL‐1β levels and escalated the LC3‐II/LC3‐I ratio. Along similar lines, Atef and colleagues evaluated simvastatin activity in an alcohol‐induced liver disease model in Swiss albino rats and observed that it restored autophagy, indicated by increased LC3‐II levels as well as to some extent, reversal of alcohol‐induced morphological damage and functional abnormalities and reduction in oxidative stress and endoplasmic reticulum stress‐induced apoptosis. Furthermore, simvastatin has been reported to ameliorate AD and reduce Aβ_40_ and Aβ_42_ load in guinea pigs [167]. In a comparative study with atorvastatin and lovastatin, simvastatin exhibited the highest reductions in Green fluorescent protein fused Aβ levels in yeast in a dose‐dependent manner [168]. However, in a phase 3 clinical trial, simvastatin did not reduce the pace of clinical progression of AD, despite reducing the body's lipid levels [169].

Gemfibrozil (aromatic ether): Rongcan Luo and colleagues were interested in whether peroxisome proliferated activated receptor‐alpha (PPARA), which has a prominent role in autophagy and metabolism of fatty acids in the liver, could be explored as a target for Aβ clearance, as in case of PP‐PSEN1/E9 mice. Gemfibrozil, an FDA‐approved antihyperlipidemic PPARA agonist, was selected for further evaluation along with another agonist Wy14643. The results from the behavioral, in‐vitro, and in‐vivo examinations revealed that the drug significantly reduced cognitive dysfunction and enhanced spatial awareness, along with a marked reduction in anxiety. The autophagosomes generated in microglia and astrocytes near the plaques were observed, which was attributed to a reduction in soluble as well as insoluble Aβ in both cortex and hippocampus tissue [128]. In another study, gemfibrozil reduced Aβ plaques and neuroinflammatory processes such as astrogliosis and microgliosis in 5XFAD mice. Improvement in spatial awareness and cognitive function was confirmed by the Barnes maze and T‐maze test [129]. Moreover, a phase‐2 clinical trial (NCT02045056) was conducted to evaluate the safety and efficacy of gemfibrozil in predementia. The double‐blind, placebo‐controlled trial reported improvement in AD‐related neuroinflammation along with a marked reduction in Aβ_42_ levels and atrophy in the hippocampus [130].

Nicotinamide (Vitamin B3): It is water soluble Vitamin B3, which is FDA‐approved as a food additive and is listed as a GRAS agent [170]. The mechanism by which nicotinamide has been proposed via PARP‐1 inhibition, DNA repair, ROS inhibition, etc [171, 172]. In recent times, its role in the induction of autophagy has become a topic of research, consequentially leading to further exploration [131]. In a study on H9C2 chronic hypoxic myocardial cells, it was found that nicotinamide treatment improved the viability of the cells, while also augmenting Atg5 (Autophagy‐ Related Gene 5) expression and LC3‐II: LC3‐I ratio [132]. Kang et al. demonstrated the induction of autophagy in fibroblast cells. They noted a significant attenuation of mitochondrial mass in nicotinamide‐treated cells via autophagy. Nicotinamide treatment enhanced the replicative lifespan of fibroblasts and improved mitochondrial quality expressed as increased membrane potential (ΔΨm). Senescent cells exhibit a reduction in autophagosome formation and autophagy, whereas ROS generation increases. This leads to over‐accumulation of damaged and disrupted cell organelles malfunctioning cell constituents and ultimately aging‐related abnormalities. Nicotinamide treatment led to augmentation in autophagic processes and increased autophagic flux [133].

In another study by Liu and colleagues on the effect of nicotinamide in AD, notable improvement in mitochondrial intactness was seen in nicotinamide‐treated 3xTgAD mice. It attenuated cognitive decline and AD‐related pathological markers, namely Aβ levels and tau hyperphosphorylation along with improved cognitive ability and spatial awareness [134]. However, a phase 2 clinical trial to evaluate the efficacy and safety of nicotinamide in mild to moderate AD failed to demonstrate any plausible efficacy of nicotinamide in the aforementioned conditions [135]. However, another phase 2a clinical trial exploring the role of nicotinamide in the modulation of early AD (NEAT study) has also been completed, with results showcased at the 2023 Alzheimer's Association International Conference. Nicotinamide attenuated changes in Clinical Dementia Rating‐Sum of Boxes (CDR‐SB) concerning placebo, with improved CSF p‐tau181 and total tau levels (Neurology Live, 2023).

Vorinostat (carboxylate): Vorinostat is an inhibitor of histone deacetylase (HDAC), FDA‐approved for cutaneous T‐cell lymphoma (CTCL) [137]. A research study conducted by Hrzenjak and colleagues validated the autophagy‐inducing potential of vorinostat on the ESS‐1 tumor cell line. There was an augmentation of mono dansyl cadaverine (MDC) levels after treatment with vorinostat, which indicated an elevated accumulation of autophagic vacuoles in the cells concerning control. Moreover, notable concentration‐dependent protein level attenuation of autophagy signaling proteins mTOR and phosphor‐mTOR provided further validation for the same [173]. Another study by Dupéré‐Richer unraveled that initial administration of vorinostat induces autophagy in parental U937 cells, however, there is a gradual adaptation, and the cells begin to utilize this augmentation in autophagy to enhance cell survival and consequentially there is a rise in vorinostat resistance, which was reversed with chloroquine [136]. To confirm the hypothesis of HDAC inhibitors inducing autophagy, Shao and colleagues studied observed Bcl‐XL overexpressing HeLa cells previously treated with vorinostat under a transmission electron microscope (TEM), which is regarded as the gold standard for examination of cell ultrastructure. Morphological alterations characteristic of autophagy‐induced cell death were observed in these cells, such as vacuolization, mutilated nuclei, autophagosome accumulation, and so forth [174].

HDAC inhibitors have been tested as a treatment option for AD in cell lines as well as in animal models, and vorinostat has been no exception. Kilgore and colleagues evaluated the efficacy of multiple HDAC inhibitors in the APPswe/PS1dE9 mouse model of AD and noted that vorinostat recovered memory functions and spatial awareness. It was also the only HDAC inhibitor to target HDAC6 [175]. Similarly, Benito et al. revealed that along with neurological improvement, vorinostat also reduces Aβ accumulation and neuronal inflammation as well as assists restoration of genetic changes [176]. However, another study reported limited bioavailability of vorinostat in the Tg2567 mouse model of AD. This was attributed to the poor pharmacokinetic profile of the drug [177], which may be improved by formulation development and optimization processes [178].

Furthermore, various combination therapies with vorinostat have also been explored for AD. Treatment with a cocktail therapy of curcumin, vorinostat, and silibinin in Aβ_25‐35_ pretreated PC12 cells attenuated ROS levels, recorded via measurement of fluorescence under confocal microscopy. Reduction in superoxide dismutase (SOD) and malondialdehyde (MDA) levels further supported this finding. The cocktail preserved Akt/MDM2/p53 signaling and also had a neuroprotective action against Aβ25‐35‐induced cellular toxicity [179]. Furthermore, to increase the therapeutic efficacy, thereby attenuating dose‐dependent toxicity of HDAC inhibitor vorinostat, a phosphodiesterase‐5 inhibitor, namely tadalafil was administered concomitantly in an APP/PS1 mice model of AD with lower doses of the former. It was observed that the drugs worked in synergism to alleviate the symptoms and improve the pathological identifiers of AD, viz cognitive dysfunction, reduced dendritic spine density, Aβ load, and tau hyperphosphorylation [180]. Augmentation of clusterin in human astrocytes by vorinostat has also been linked with its anti‐AD activity [181]. Currently, there is an ongoing phase1b trial (NCT03056495) of vorinostat in AD [144].

## 7 Drugs in Clinical Trials for AD With Linked Autophagy‐Inducing Mechanism

Due to the limited therapeutic options for AD, a wide array of drugs is being currently explored for their safety and efficacy in the aforementioned condition. Several of these are currently in clinical trials (as mentioned in Table 3) for AD therapy, some of which have linked autophagy‐inducing mechanisms as well. For example, tyrosine kinase inhibitors such as nilotinib and dasatinib are currently undergoing clinical trials for AD [106]. As discussed above, these moieties have autophagy‐inducing capabilities. Several polyphenols such as quercetin, resveratrol, and curcumin, which have autophagy‐inducing capabilities, are also currently undergoing phase II clinical trials for AD (NCT06470061) [202]. Rapamycin and niacin, which induced autophagy as discussed above, are currently undergoing phase I clinical trials for AD (NCT05723172) (NCT06582706).

**Table 3:** Drugs in clinical trials for AD with a linked autophagy‐inducing mechanism that has the potential to modulate autophagy via different targeting pathways are summarized.

Drug	Class	Conventional use	Linked autophagy‐inducing mechanism
Nilotinib	Benzamide	Tyrosine kinase inhibitor	AMPK activation, HDAC inhibition
Trehalose	Carbohydrate	Food additive	mTOR independent pathway [182], TFEB activation [183]
Mirtazapine	Tetracyclic antidepressants	Inhibitor of presynaptic alpha‐2‐adrenergic receptor	HMGB1‐mediated autophagy [184]
Rapamycin	Macrocyclic drug	Immunosuppressant (mTOR inhibition)	mTOR inhibition mediated [185]
Hydralazine	hydrazinophthalazine	Vasodilation	Not known [186]
Levetiracetam	pyrrolidine	Antiepileptic	AMPK activation [187]
Siponimod	Sphingosine‐1‐phosphate receptor modulator	Sphingosine 1‐phosphate receptor (S1P) modulator	Not mentioned [188]
Vortioxetine	Piperazine derivative	Serotonin reuptake inhibitor	PI3K/AKT pathway [189]
Niacin	Vitamin B3	pellagra	↑LC3‐II, ↓p62 [190]
Foralumab	Anti CD3 antibody	T cell modulation	Upregulation of autophagy‐linked genes [191]
Curcumin	Polyphenol	Anti‐inflammatory	PI3K/Akt/mTOR signaling pathway
Resveratrol	Polyphenol	Anti‐inflammatory	Mitophagy and AMPK/mTORC1 mediated [22, 23]
Quercetin	Flavonoid	Anti‐inflammatory	↑Atg5, Atg12, Atg16L, Lc3B, and Beclin1 [192]
Dasatinib	Benzamide	Tyrosine kinase inhibitor	AKT/mTOR mediated [107]
Dronabinol	cannabinoid	HIV/AIDs‐induced anorexia	PI3K/AKT pathway [193]
Formoterol	Formamide	long acting β₂ agonist	SQSTM1/p62‐dependent [194]
Sargramostim	Glycoprotein	Recombinant human granulocyte‐macrophage colony‐stimulating factor (rhGM‐CSF)	↑autophagosome [195]
Pimavanserin	Piperidine derivative	Serotonin receptor blocker	ULK1‐mediated autophagy [196]
Roflumilast	benzamide	Phosphodiesterase 4 inhibitor	AMPK/mTOR/ULK1‐dependent autophagy [197]
Idazoxan	Dioxane	α2‐adrenoceptor inhibitor	↑LC3‐II [198]
Blarcamesine	Diphenyl oxolane	SIGMAR1 agonist	SIGMAR1 mediated [199]
Methylphenidate	Phenethylamine	Norepinephrine and dopamine reuptake inhibitor	↑Beclin‐1, LC3B [200]
Bryostatin	Macrocyclic lactones	Protein kinase C activation	mTOR mediated [201]

Trehalose is a carbohydrate commonly used as a food additive in various food processing industries [203]. Chen et al., in their research, have unraveled that trehalose as well as other carbohydrates such as sucrose and raffinose induce autophagy by augmenting the conversion of LC3‐I to LC3‐II via an mTOR‐independent pathway [203, 204]. Mirtazapine is an antidepressant that acts via inhibition of adrenergic α_2_‐autoreceptors and α_2_‐heteroreceptors as well as 5‐HT_2_ and 5‐HT_3_ receptors [205]. It has been found to induce autophagy, however, induction of autophagy by mirtazapine was observed to be via HMGB1‐dependent Akt/mTOR pathway, and induced cardiotoxicity in a C57BL/6 J mice model, which was further augmented by alcohol [184]. Further research is required to warrant its use as a therapeutically active autophagy inducer.

A potent vasodilator hydralazine is conventionally used for its antihypertensive properties. While not much is reported about its role in autophagy, Mirzaei et al. have mentioned that it induces autophagy at therapeutically active doses such that it enhances the clearance of protein aggregates [186]. Levetiracetam is a potent antiepileptic that has been reported to upregulate autophagy‐promoting proteins such as beclin‐1 and LC3‐II/LC3‐I ratio in both SH‐SY5Y and APPswe/PS1dE9 transgenic mice amounting to reduced cognitive disability in mice in the preclinical setting [187]. Sphingosine‐1‐phosphate receptor modulators have recently been approved for various pathologies such as multiple sclerosis and ulcerative colitis [206]. Gruchot et al. observed that autophagy was one of the various mechanisms that got enriched upon treatment of microglia with siponimod for their activation with lipopolysaccharide [188]. Vortioxetine, a serotonin reuptake inhibitor, has been observed to induce autophagy in AGS gastric cancer cells via PI3K/AKT pathway, augmenting the levels of beclin1 and LC3 [189]. In another recent study by Lopez et al., CD‐3 monoclonal antibody Foralumab exhibited the potential to upregulate autophagy‐inducing genes in a 3xTg mice model of AD [191].

β2‐agonist administration has been known to induce skeletal muscle hypertrophy. Joassard et al. observed that β2‐agonist formoterol, when administered in rat skeletal muscle, led to an increase in autophagy gene LC3b and gamma‐aminobutyric acid receptor‐associated protein‐like 1 (Gabarapl1) [207]. In a cljnical setting of PD, Abdelmoaty et al. by proteomic analysis observed that in PD patients, treatment with sargramostim, a recombinant human granulocyte‐macrophage colony‐stimulating factor, augmented the levels of autophagy‐inducing proteins ATG3, ATG7, and GABARAPL2 within 6 months of therapy with sargramostim [208]. Pimavanserin is an atypical antipsychotic conventionally used to manage symptoms of psychosis such as delusions and hallucinations. However, a recent study by Ramachandran et al has demonstrated that it may also be used as an inducer of autophagy for pancreatin cancer therapy [196] since it augments the levels of autophagy in pancreatic ducal adenocarcinoma (PDAC) cells. The same was then demonstrated in‐vivo via a Subcutaneous xenograft pancreatic tumor model.

A phosphodiesterase‐4 inhibitor roflumilast was inspected preclinically for its therapeutic effects on ovariectomy‐induced depression‐like symptoms. It was observed that roflumilast attenuated depression‐like symptoms via induction of autophagy by activation of AMPK/mTOR/ULK1‐dependent autophagy pathway in Swiss albino rats. A consequent enhancement of p‐AMPK, p‐ULK1, Beclin‐1, and LC3II/I expression and reduction in p62 and p‐mTOR protein expression was also noted [197]. In another study, idazoxan, an imidazoline, was found to induce autophagy and augment levels of autophagy marker LC3‐II in RAW.264.7 cells [198].

ANAVEX2‐73 (blarcamesine) is a Sigma Receptor (SIGMAR1) agonist. Recently, SIGMAR1 activity has been explored for its possible implication in various cognitive and neurological conditions [199]. Along similar lines, in a study by Christ et al., ANAVEX2‐73 was demonstrated as an autophagy‐inducing agent in HeLa and HEK293A cells as well as in *C. elegans* [209]. Methylphenidate, a CNS stimulant approved for ADHD, has exhibited autophagy‐inducing capabilities in a study by Kong et al. However, the aforementioned drug has been linked with an increased risk of damage to the retina, and autophagy was observed to be a causative factor due to its negative effects on photoreceptor cells [200]. An orphan drug Bryostatin, has been approved by the FDA for the therapy of fragile X syndrome [210] has shown promise as an autophagy‐inducing agent, enhancing the autophagic flux of Aβ protein aggregates in hippocampal neurons [201].

## 8 Other Miscellaneous Autophagy Inducers

Autophagy is an important physiological process quintessential for the normal functioning of cells, promoting clearance of senescent or unessential cellular material and synthesis of new proteins and material required for various cellular processes. This section discusses various novel and conventional therapeutic moieties as autophagy‐inducers (also mentioned in Table 4) by various research groups.

**Table 4:** Other miscellaneous compounds as autophagy inducers are summarized below.

Drug	Class	Conventional use	Autophagy inducing mechanism
Spermidine	Polyamine	Supplement	Caspase‐3‐dependent Beclin‐1 cleavage [211]
Lithium	Metal	Bipolar disorder	IMPase and IPPase inhibition mediated [212]
Metixene	Thioxanthene	Parkinson's disease	NDRG1 phosphorylation induced [213]
Compound 12	Pyridine derivative	Novel compound	Not mentioned [214]
10 P	Urea derivative	Novel compound	↑ATG5 and ATG7 [215]
Albendazole	Benzimidazole	Anthelmintic	AMPK/ULK1 mediated [216]
Teniposide	Podophyllotoxin derivatives	Anticancer	JIP4‐ dependent lysosomal modulation dependent [217]
Amsacrine	Acridine derivative	Anticancer	SIDT2 mediated [218]
ABT‐263	Phenylpiperazine derivative	Anticancer	mtDNA‐STING mediated [219]
Etoposide	Podophyllotoxin derivative	Anticancer	Wip1/PPM1D mediated [220]
Oxibendazole	Benzimidazole derivative	Anthelmintic	JIP4‐ dependent lysosomal modulation dependent [217]
Mebendazole	Benzimidazole derivative	Anthelmintic	↑LC3B [221]
Microcolin H	Marine lipopeptide	Autophagy inducer	PITPα/β mediated [222]
NR1	Ginsenoside	Traditional Chinese medicine	PI3K/Akt/mTOR mediated [51]
DL001	Rapamycin analog	mTOR modulator	mTORC1 mediated [223]
NV‐5440	Sulfonylurea	mTOR modulator	mTORC1 mediated [224]
Exendin‐4	Peptide hormone	Type‐1 diabetes	PI3K/Akt/mTOR mediated [225]
Apelin‐13	Peptide hormone	Apelin receptor ligand	AMPK activation [226]

Spermidine is a known autophagy inducer with neuroprotective effects. It induces autophagy by cleavage of Beclin‐1, which is supported by the translocation of the caspase‐3 enzyme in staurosporine‐treated PC12 cells and cortical neurons [211]. Lithium is an approved medication for bipolar disorder. It has shown promising preclinical results for its therapeutic application in other neurological disorders, including AD [227]. A couple of studies have reported autophagy‐inducing effects of lithium, via inhibition of Inositol monophosphatase (IMPase) and inositol polyphosphate‐1‐phosphatase (IPPase), which further downregulates IP_3_‐DAG levels via attenuation of inositol levels [212, 228].

Metixene, an anticholinergic drug used in the therapy for PD, has been observed to induce autophagy preclinically in metastatic cancer and brain metastasis in immunodeficient mice models and BT‐474Br and MDA‐MB‐231 cancer cells. The moiety was found to induce incomplete autophagy via N‐Myc downstream regulated 1 (NDRG1) phosphorylation which subsequently caused caspase‐induced apoptosis in the cells [213]. A recent study by Shaban et al. focuses on synthesizing novel pyridine derivatives with profound apoptosis and autophagy‐inducing capabilities on MCF‐7 Cells. They observed that a novel molecule ‘Compound 12’ exhibited profound autophagy‐induced cell death, which was analyzed through flow cytometry using acridine orange lysosomal dye. The IC_50_ value for the same was 0.5 and 5.27 μM for MCF‐7 and HepG2 cell lines respectively [214]. Another research group Liang et al. designed and synthesized various urea derivatives 10a ∼ 10r, 11a, 11b. and 12a ∼ 12c and examined for their autophagy‐inducing properties. The results suggested that the 10p was most potent with IC_50_ values of 1.97 ± 0.01, 6.18 ± 0.56, 4.92 ± 0.61, 7.01 ± 2.23, and 13.79 ± 3.15 against HT‐29, HeLa, K562, MCF‐7, and HEPG2 cells respectively It was also observed that 10p caused cell cycle arrest at G2/M phase [215].

Drugs with antiparasitic activity such as albendazole and mebendazole have been conventionally used for their anthelminthic activity. A recent study by Jung et al. centered around the autophagy‐inducing potential of albendazole has revealed that it induces AMPK and ULK1 phosphorylation‐mediated autophagy in human colon adenocarcinoma cells such as HCT‐15, HCT‐116, HT‐29, and SW480 cells [216]. Similarly, mebendazole was found to induce autophagy in HUVEC cells. An increase in LC3B+ puncta in HUVEC cells at 1 and 3 μM concentrations was observed via immunofluorescence, further validated using siRNAs of ATG5, ATG7, and Beclin1 to knock out the specific autophagy mediating genes [221]. In a separate study, oxibendazole and teniposide, a podophyllotoxin derivative, were observed to induce autophagy via lysosomal co‐localization augmented by TMEM55B‐ JNK‐ interacting protein 4 (JIP‐4) in SH‐SY5Y cells and extensively verified using JIP‐4 knockout cells [217]. A couple of other anticarcinogenic agents, amsacrine and etoposide have also preclinically exhibited autophagy‐inducing activity. Lee et al. in a recent study discovered that amsacrine induces autophagy in human chronic myeloid leukemia (CML) cells by augmentation of SIDT2 levels, subsequently leading to attenuation of miR‐25. This reduction in miR‐25 expression consequently led to an aggravated NOX4‐mediated ROS production [218]. Also, ABT‐263 (Navitoclax) an experimental BCL2 inhibiting anticancer molecule had autophagy‐inducing properties, mediated by increased IKKα/β‐NFκB axis expression in U937 cells, with cell viability being diminished to 50% after 24 h of 1 μM treatment with ABT‐263 [229]. The autophagic nature of ABT has been further validated through various other studies [219]. The compound has also preclinically been able to promote the clearance of senescent glial cells, warranting further research as a therapy for AD as well [230]. The response of DNA‐damaging anticancer agents on autophagy was examined by researchers at the University of Bern, where they discovered that even nonlethal microdoses of agents such as cisplatin and etoposide were able to trigger the augmentation of ATG5, a prominent mediator of autophagy, causing mitotic catastrophe, independent of induction of autophagy. However, a separate study by Karlitepe et al. and Pilevneli et al. demonstrated that etoposide markedly induces serin/threonine phosphatase (Wip1/PPM1D) mediated autophagy in MCF‐7, D283‐med and IMR32 cells, with rise in LC3I/II and p62 expression, analyzed via western blot and immunostaining assays [220, 231].

Microcolins, obtained from marine cyanobacterium *Moorea producens*, are structurally identical to lipopeptides and have anticancer properties. Its anticancer effects were attributed to its modulation of phosphatidylinositol transfer protein alpha/beta isoform (PITPα/β) and triggers autophagy leading to an increase in the levels of LC3II to LC3I and Beclin‐1. However to ensure its role in AD, further research is needed [222].

Certain mTORC modulators such as NR1, DL001, and NV‐5440 have also been explored as autophagy inducers and subsequently exhibited preclinical prowess as the same [232]. NV‐5440 was observed to have an IC_50_ value of 0.07 μM in MCF7 cells [224], whereas DL001 had an IC_50_ value of 74.9 pM in PC12 cells [223]. Specific peptides such as Exendin‐4, apelins, and myokines also have been reported as autophagy inducers. Exendin‐4 has been recently analyzed for its role in the modulation of autophagy in an AAV‐9‐A53T‐α‐synuclein‐administered animal model of PD. It was discovered that treatment with exendin‐4 diminished the pathological α‐synuclein and consequently attenuated neuronal degradation and improved cognitive ability [225]. Apelin‐13 is another such peptide that has exhibited bone protection potential in bone marrow mesenchymal stem cells and OVX rats via activation of mitophagy [226]. Gemfibrozil, a phenolic ether conventionally used as a lipid‐lowering agent, is a PPARA agonist. It has been earlier reported and validated that PPARA activation through an agonist potentiates autophagy pathways in cells [233]. Wy14643, an agonist of PPARA receptors, has been studied for its effects on autophagy by Luo et al. both in vitro in human microglia (HM) cells and U251 human glioma cells expressing mutant APP‐p.M671L and in vivo in APP‐PSEN1ΔE9 mice. They observed a marked reduction in both soluble and insoluble Aβ protein in hippocampal and cortical regions, along with increased microglial autophagosome expression [128].

## 9 Future Perspectives and Conclusion

Autophagy is a complex biological process that plays a dual role in health and disease. Its manipulation, either stimulation or inhibition, has proven beneficial depending on the disease context. In cancer, autophagy can diminish tumor growth by clearing senescent cells and toxic metabolites, but it can also be exploited by cancer cells for survival under stress [234]. In neurodegenerative diseases like Alzheimer's disease (AD), Parkinson's disease (PD), and Huntington's disease (HD), impaired autophagy contributes to the accumulation of misfolded proteins and cellular debris, exacerbating neurodegeneration. In AD specifically, reduced autophagy hampers the clearance of amyloid‐β (Aβ) plaques and tau protein, leading to progressive cognitive decline and memory loss [235, 236]. This culminates in progressive memory loss, mental confusion, forgetfulness, and eventually, inability to perform daily tasks and follow basic instructions [17].

Current FDA‐approved AD treatments include cholinesterase inhibitors (donepezil, rivastigmine, and galantamine), and NMDA receptor antagonists (memantine) [237], and monoclonal antibodies (aducanumab and lecanemab), primarily address symptoms or reduce amyloid load. Aducanumab and Lecanemab exhibit side effects like Amyloid Imaging Abnormalities (ARIA), headache, and delirium, which can be fatal in some cases, therefore limiting their use in patients [238].

Various autophagy inducers have been investigated for their therapeutic potential in Alzheimer's disease (AD). Some candidates have progressed to clinical trials, while others have demonstrated improvements in memory and cognitive functions in animal models. However, further research is essential to achieve the ultimate therapeutic goal for AD. Since current pharmacological treatments primarily focus on symptom management, there is an urgent need for disease‐modifying therapies. Autophagy inducers hold significant promise as future treatments for AD.

Many of these promising inducers belong to BCS Class 2 or Class 4, presenting challenges such as low bioavailability, limited permeability, and off‐target adverse effects. Nanotechnology offers innovative solutions to these limitations by facilitating targeted drug delivery, improving drug solubility and stability, and enhancing the ability of drugs to cross the blood‐brain barrier (BBB). These advancements optimize therapeutic efficacy while minimizing systemic side effects, addressing the inherent drawbacks of these drug classes [239]. Nanoparticle‐based strategies have opened new avenues for realizing the full therapeutic potential of autophagy inducers. By enhancing bioavailability and enabling precise delivery to target tissues, these approaches reduce dosing frequency and significantly lower the risk of off‐target side effects, thereby improving the safety and effectiveness of autophagy‐inducing therapies [240, 241, 242]. Continued exploration of autophagy‐based therapies, combined with nanotechnology, could lead to breakthroughs in AD treatment, expanding therapeutic options and addressing unmet needs. With further research, autophagy inducers might emerge as a cornerstone of future AD pharmacotherapy, moving beyond symptomatic relief to modifying the course of the disease.

## Author Contributions

Conceptualization was carried out by Baljinder Singh, Shubham Mahajan, Rehan Khan, and Sanjay Garg; the original draft was prepared by Baljinder Singh and Shubham Mahajan; review and editing were performed by Baljinder Singh, Shubham Mahajan, Sadikalmahdi Abdella, Rehan Khan, and Sanjay Garg; supervision was provided by Sanjay Garg and Rehan Khan. All authors have read and approved the final version of the article.

## Conflicts of Interest

The authors declare no conflict of interest.

## Data Availability

The authors have nothing to report.
